# Quantum Chemical Characterization and Design of Quantum Dots for Sensing Applications

**DOI:** 10.1021/acs.jpca.2c00947

**Published:** 2022-05-03

**Authors:** Aleksandra Foerster, Nicholas A. Besley

**Affiliations:** School of Chemistry, University of Nottingham, University Park, Nottingham NG7 2RD, U.K.

## Abstract

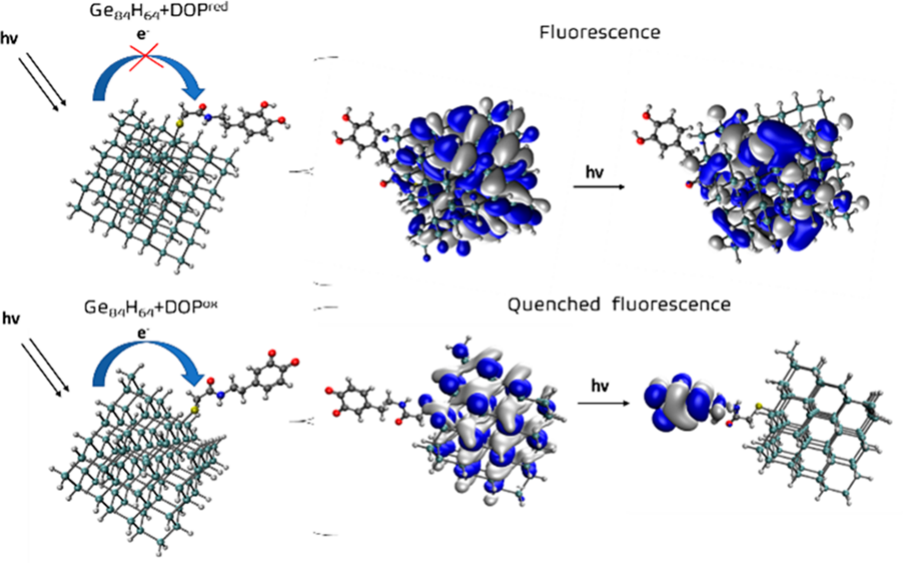

The ability to tune the optoelectronic properties of quantum dots (QDs) makes them ideally suited for the use as fluorescence sensing probes. The vast structural diversity in terms of the composition and size of QDs can make designing a QD for a specific sensing application a challenging process. Quantum chemical calculations have the potential to aid this process through the characterization of the properties of QDs, leading to their *in silico* design. This is explored in the context of QDs for the fluorescence sensing of dopamine based upon density functional theory and time-dependent density functional theory (TDDFT) calculations. The excited states of hydrogenated carbon, silicon, and germanium QDs are characterized through TDDFT calculations. Analysis of the molecular orbital diagrams for the isolated molecules and calculations of the excited states of the dopamine-functionalized quantum dots establish the possibility of a photoinduced electron-transfer process by determining the relative energies of the electronic states formed from a local excitation on the QD and the lowest QD → dopamine electron-transfer state. The results suggest that the Si_165_H_100_ and Ge_84_H_64_ QDs have the potential to act as fluorescent markers that could distinguish between the oxidized and reduced forms of dopamine, where the fluorescence would be quenched for the oxidized form. The work contributes to a better understanding of the optical and electronic behavior of QD-based sensors and illustrates how quantum chemical calculations can be used to inform the design of QDs for specific fluorescent sensing applications.

## 1 Introduction

Biosensors play a vital role in medicine, where, for example, early detection of cancer biomarkers can significantly reduce morbidity and mortality worldwide.^1,2^ Optical biosensors form one class of biosensors and can be applied to the detection of disease biomarkers and other biomolecules.^3−5^ An optical biosensor is often comprised of different components that have different functions. The receptor is responsible for capturing the molecule (analyte) under study, which leads to a detectable change in the fluorescence of the fluorophore, in the form of a change in wavelength or an enhancement or quenching of the fluorescence. A wide variety of chemical species can be used as the fluorophore, ranging from bodipy dyes^6,7^ to green fluorescent protein.^8−12^ More recently, the application of quantum dots (QDs) in biosensing has become increasingly important^8−10^ owing to their favorable sensing properties, biocompatibility, and chemical inertness.^13^

QDs are 1–20 nm luminescent semiconductor nanostructures with interesting photophysical and photochemical properties.^14−17^ The small size of QDs gives them unique absorption and emission properties as a consequence of the quantum confinement effect leading to their photoluminescence being size- and composition-dependent.^15^ Through variation in composition and size, it is possible to obtain fluorescence in the full spectral range from ultraviolet (UV) to near-infrared coupled with the capability to tune the fluorescence. They are characterized by a broad absorption band and a narrow fluorescence emission profile, which makes them ideal candidates for optical sensor applications. Additionally, QDs can be repeatedly excited without a noticeable decrease in their fluorescence because they have a high quantum yield, as well as a long radiation emission time (10–100 ns). Moreover, their fluorescence shows high resistance to photobleaching.^18,19^

Some of the examples of using QDs in biosensors include manganese-doped zinc sulfide (ZnS:Mn/Zns) quantum dots functionalized with cysteine for the sensing of bilirubin,^20^ thioglycolic acid-functionalized ZnS:Mn/ZnS quantum dots for the sensing of creatinine,^21^ and CdSe/ZnS quantum dots functionalized with dopamine (DA) for the sensing of the α-fetoprotein.^22^ A number of different photophysical processes underpin the sensing mechanisms in QD-based systems, and these include photoinduced electron transfer (PET), charge transfer (CT), and fluorescence resonance energy transfer (FRET).^23−25^ In the case of the PET mechanism, the excited fluorophore can accept or donate electrons leading to quenching of the photoluminescence in oxidative or reductive PET processes.^25^ For the CT mechanism, there is a change in the overall charge distribution in the system. Often the receptor and fluorophore coexist in the same conjugated system or there is a strong interaction between them making the photophysical behavior strongly dependent on the analyte.^23^ In the FRET mechanism, a transfer of excitation energy occurs causing a decrease in the donor photoluminescence intensity, and the distance between the donor and acceptor can influence the fluorescence process.^24^

Alongside experimental work,^26,27^ computational studies can contribute to the understanding of the relationship between the structural and optical properties of QDs. Furthermore, they allow the nature of the sensing mechanism to be probed and have the potential to guide the design of QDs for specific sensing applications. In recent years, there has been a number of studies that have used density functional theory (DFT) and time-dependent density functional theory (TDDFT) to study the properties of fluorescent biosensors^28−30^ including QD-based systems.^31−37^ It is common to interpret the photophysical behavior of the fluorophores in terms of the molecular orbital diagram based upon the Kohn–Sham orbitals and energies.^38−43^ A more accurate approach is to calculate the excited states explicitly through TDDFT or higher-level wavefunction-based calculations, which can also provide greater insights into the sensing mechanisms of the fluorescent probes.^44−53^ Examples of studies of QDs include hydrogenated silicon quantum dots of varying sizes,^32^ boronizated and oxidated graphene QDs,^33^ and graphene QDs functionalized with various oxygen-containing functional groups.^33^ Calculation of emission spectra has been reported for functionalized graphene quantum dots,^35^ acetate-functionalized CdSe,^36^ or halogen atom-passivated silicon nanocrystals.^37^ Dye-sensitized QDs have been studied with TDDFT with the effects of solvent included through polarized continuum solvent models.^54,55^ Also, studies involving orbital characterization have been performed for ligated QDs, which have included investigation of CT mechanisms.^25,56,57^

The studies discussed above demonstrate that it is possible to use DFT and TDDFT calculations to characterize the properties of QD-based sensors and rationalize the photophysical processes underlying the sensing mechanism. This leads to the question of whether these calculations can be utilized in the design of sensors for a particular sensing application. In this work, we focus on a dopamine (DA)-functionalized QD sensor, similar to one reported in experimental work by Zhang et al.^22^ This sensor detects the disease biomarker α-fetoprotein exploiting the catalytic oxidation of DA to DA-quinone, which results in the fluorescence quenching of a CdSe/ZnS QD. We have explored how DFT and TDDFT calculations can be exploited to characterize and guide the design of hydrogenated carbon silicon and germanium QDs for this sensing application.

## 2 Evaluation of Computational Methods

**Figure 1:**
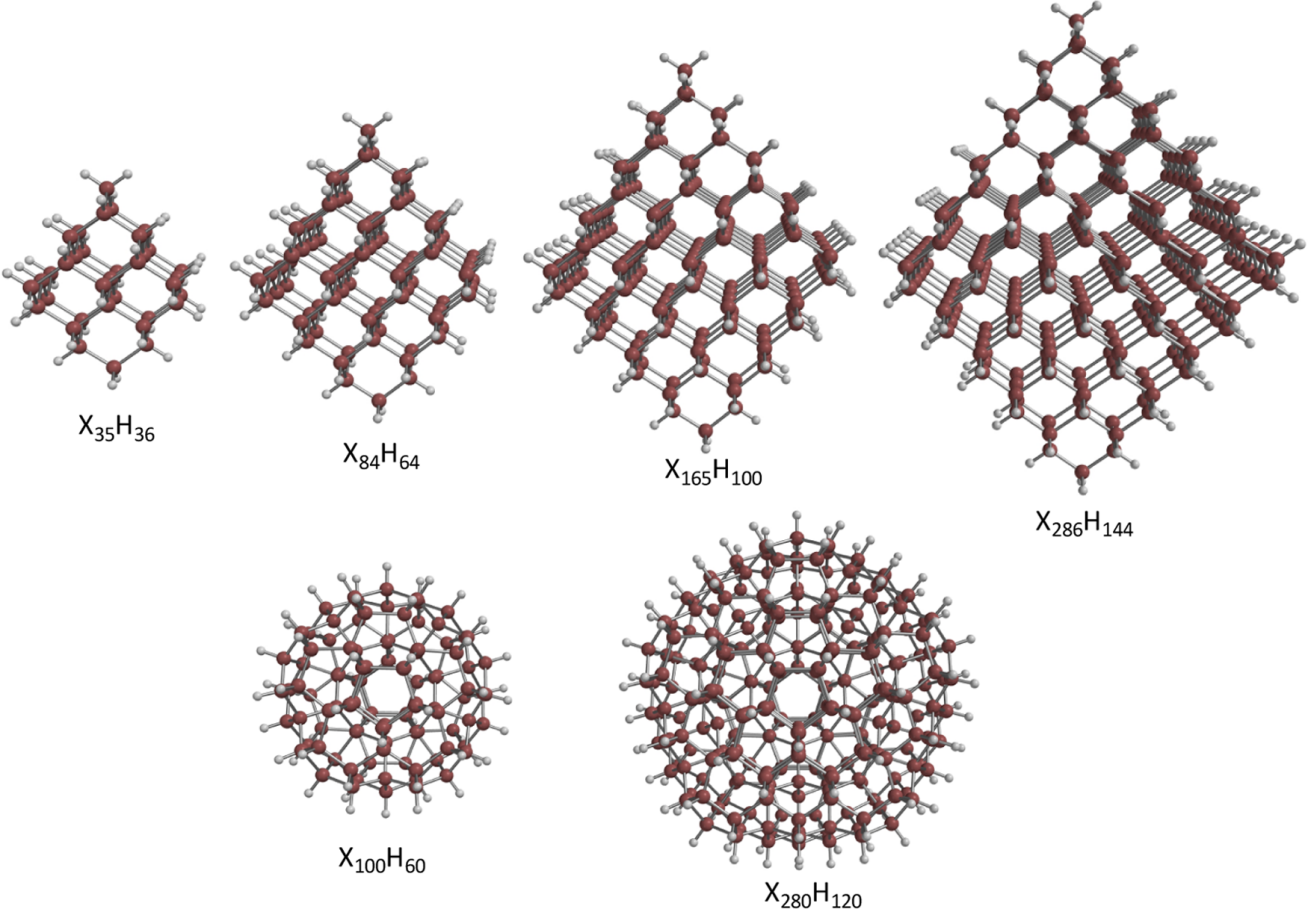
Structures for QDs with various sizes, X = Ge, Si, or C.

The structure of the QDs was taken from the work of Karttunen et al.^58^ who studied the structure and electronic properties of a series of hydrogenated carbon, silicon, and germanium clusters using DFT with the B3LYP functional^59^ and def2-SVP basis set. It was found that the band gap decreases as the size of the cluster increases. The band gaps for the silicon and germanium clusters are similar and smaller than those of the carbon clusters of comparable size. In this work, two types of QDs have been studied, those with diamond-like structure and icosahedral structure, and these QDs are shown in Figure 1. The size of the QDs ranges from 35 to 280 C, Si, or Ge atoms with 36 to 120 H atoms. The diameter of the small (X_35_H_36_) QDs is 0.9 nm for Si, 1.3 nm for Ge and C, and for the large (X_280_H_144_) QDs 3.1, 2.9, and 1.9 nm for Ge, Si, and C, respectively.^58^ The structures of the QDs with the oxidized and reduced forms of dopamine absorbed onto the QDs were optimized using the B3LYP/def2-SVP level of theory. The initial choice of the attachment of the DOP molecule to the QD surface via −(C=O)CH2S linking group was guided by experimental observations.^22^ The effect of the anchoring group and position of DOP on the QD surface (edge, corner, face sites) has been further investigated.

To be able to study the larger QDs, it is necessary to establish a level of theory that is both computationally efficient and accurate. The excited states and corresponding absorption spectra were first computed using TDDFT with the CAM-B3LYP functional^60^ and a range of all-electron and effective core potential (ECP) basis sets (Table 1). All calculations were performed using the Q-Chem software package.^59^ In the Supporting Information (Table S1), the excitation energies calculated with the Tamm–Dancoff approximation (TDA) are also included for completeness; however, these calculations show that in all cases of interest the TDA approximation does not improve the values of excitation energies. Here, we focus on the low-lying excited states since these are the most relevant for a fluorescence-based sensor. There have been relatively few computational studies of the excited states of these systems, but some TDDFT calculations primarily for silicon have been reported^32,61−63^ and TDDFT calculations have been shown to be accurate for studying other systems composed of dopamine and nanoparticles such as TiO_2_,^64^ Ag,^65^ and Au.^66^

**Table 1:** Lowest Four Singlet Transition Energies (Δ*E*, eV) for C_35_H_36_, Si_35_H_36_, and Ge_35_H_36_ Quantum Dots Calculated Using TDDFT/CAM-B3LYP with a Range of Basis Sets

Δ*E* (eV)						
	6-31G*	6-31+G*	6-31++G**	def2-SVP	LANL2DZ	SRLC
Ge_35_H_36_	4.87	4.84	4.84	4.90	5.20	4.87
	5.03	5.00	5.00	4.93	5.34	5.04
	5.09	5.04	5.03	5.09	5.41	5.14
	5.17	5.12	5.10	5.20	5.42	5.25
Si_35_H_36_	5.17	5.19	5.18	5.16	5.68	5.54
	5.26	5.20	5.19	5.27	5.78	5.64
	5.28	5.39	5.38	5.30	5.83	5.70
	5.48	5.46	5.45	5.50	6.08	5.87
C_35_H_36_	8.17	6.45	6.30	7.62	8.14	8.14
	9.18	6.89	6.71	8.43	9.13	9.15
	9.47	7.23	7.06	8.80	9.47	9.49
	9.52	7.29	7.13	8.85	9.53	9.53

Table 1 summarizes the lowest four singlet transition energies for X_35_H_36_ QDs (X = C, Si, Ge) calculated using the TDDFT/CAM-B3LYP level of theory with 6-31G*, 6-31G+*, 6-31++G**, and def2-SVP all-electron basis sets and LANL2DZ and SRLC ECP basis sets. We note that in the latter case, the 6-31G basis set is used for the hydrogen atoms. For Ge- and Si-containing quantum dots, the results show a weak variation of the predicted transition energies with the choice of all-electron basis sets; however, the effect is significant for the C-based QDs. For C_35_H_36_, adding polarization functions leads to an improvement in the accuracy of predictions for singlet transition energies as compared to the results obtained with the ECP basis sets. Therefore, further calculations on larger C-containing QDs were performed using the 6-31+G* basis set.

The performance of the selected ECP basis sets is variable. In all cases, LANL2DZ values overestimate the transition energies predicted with all-electron basis sets, although for the larger SRLC basis set the values for Ge_35_H_36_ are in good agreement with those for 6-31G*, 6-31+G*, 6-31++G**, and def2-SVP. There remains some discrepancy in predictions for Si_35_H_36_, but the agreement is sufficiently close to justify the use of the SRLC basis set in calculations for larger Ge- and Si-containing QDs where the size difference between ECP and all-electron basis sets becomes significant (for the Ge_165_H_100_ cluster, the SRLC basis set employs 1520 basis functions compared to 5480 basis functions of the 6-31G* basis set). Additionally, a similar comparison was produced for the lowest singlet transition energies in functionalized X_35_H_36_ QDs with attached oxidized and reduced forms (Table S2 in the Supporting Information) to support the above conclusion regarding the use of SRLC basis for bigger systems.

## 3 Results and Discussion

### 3.1 Characterization of the QDs and Dopamine

**Figure 2:**
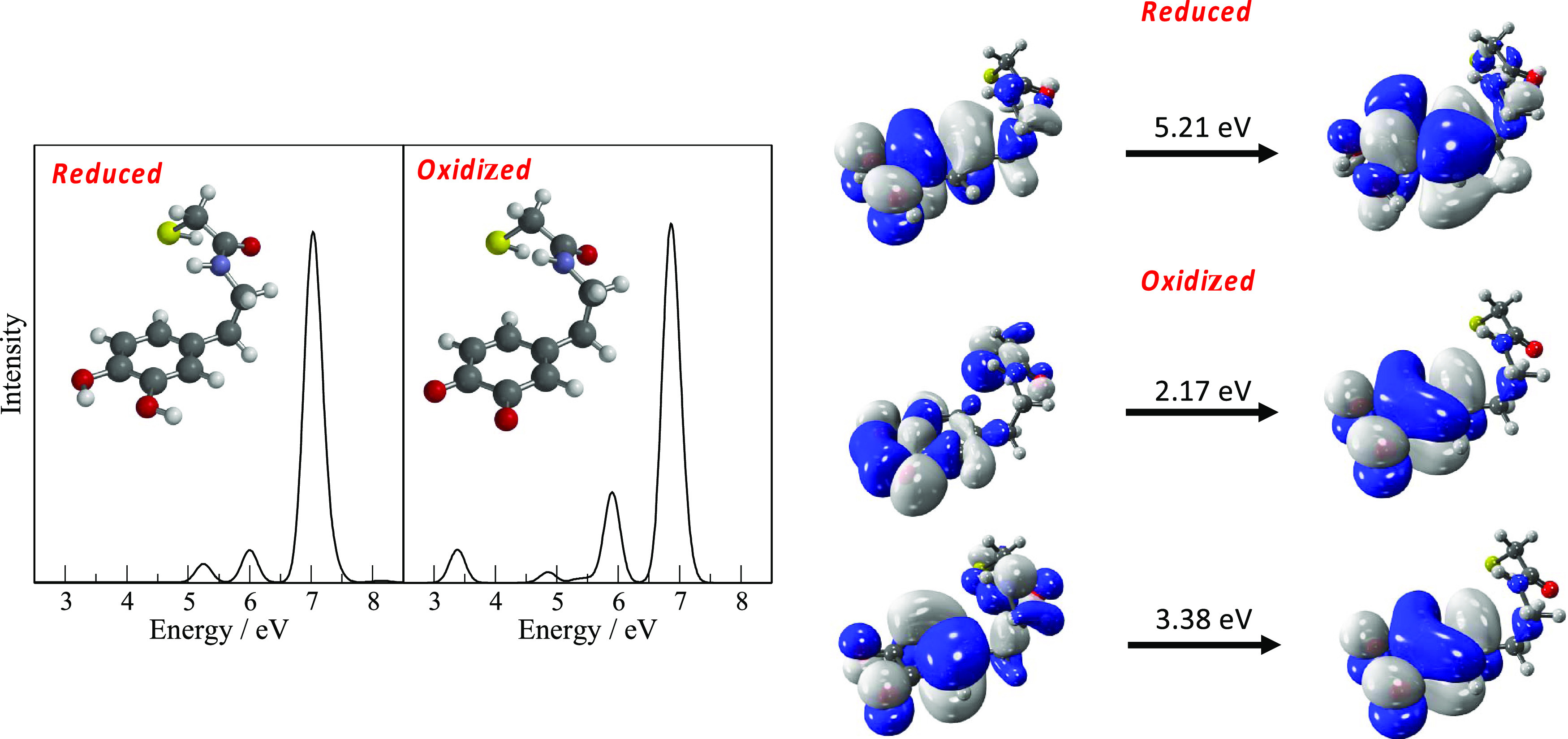
Absorption spectra for oxidized and reduced forms of dopamine with the linking group, and the orbitals associated with the lowest-energy transitions (excitations below 3 eV are not visible).

Absorption spectra were generated by convoluting the calculated excitation and oscillator strengths with Gaussian functions with a full-width half-maximum of 0.3 eV. Figure 2 shows the computed TDDFT CAM-B3LYP/SRLC electronic spectra for the oxidized and reduced forms of dopamine. In these calculations, the −(C=O)CH_2_S- group used to attach dopamine to the quantum dot is included. The absorption spectra and the excitation energies for dopamine with and without the inclusion of the linking group are included in the Supporting Information (Figure S1) to show that the lowest-energy transitions are associated with the dopamine part of the molecule, while the linking group contributes to the transitions at higher energy. Also shown in the Supporting Information are the calculated excitation energies with and without the TDA. These show that the introduction of the TDA does have a noticeable effect on some of the calculated excitation energies and it was decided that this approximation would not be used in the subsequent analysis. The spectra also reveal the critical difference between the two forms of dopamine where the lowest-energy transitions for the oxidized form are significantly (∼3 eV) lower in energy compared to the reduced form. The orbitals associated with the lowest-energy transitions are also shown in Figure 2 (isovalue of 0.02 A^–3^ is used to produce isodensity plots). These are predominantly on the dopamine part of the molecule and consequently will be affected by the change from OH to =O substituents on the ring.

**Figure 3:**
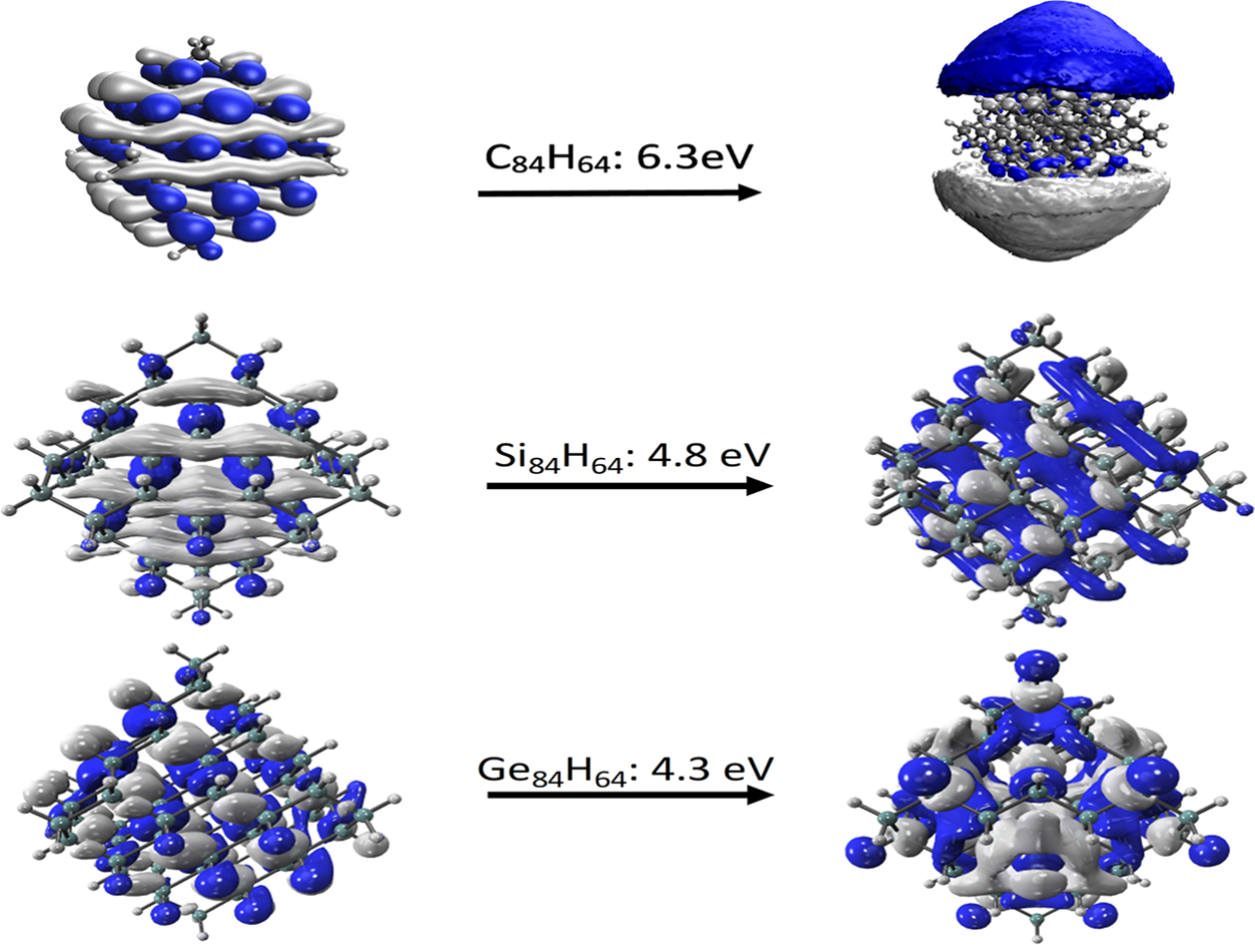
Molecular orbitals associated with the intense transition for the X_84_H_64_ QDs.

Table 2 summarizes values for three important quantities, namely, the highest occupied molecular orbital–lowest unoccupied molecular orbital (HOMO–LUMO) gap (Δ*E*_HL_), the excitation energy for the lowest singlet state (Δ*E*_S1_), and Δ*E*_MAX_, which is the excitation energy for the most intense transition, within the energy range studied, calculated at the TDDFT CAM-B3LYP level of theory (SRLC basis set was used for Si- and Ge-based QDs and 6-31+G* for C-containing QDs). For the largest clusters, it was not possible to obtain values for Δ*E*_MAX_. The results show that the excitation energy for the lowest singlet state decreases as the size of the cluster increases and this observation is consistent with previous studies.^58,67,68^ The value of the excitation energy for clusters of a similar size also decreases going from carbon → silicon → germanium, and there is a large difference between the carbon-based QDs and the silicon and germanium ones. For example, for the X_84_H_86_ QD, Δ*E*_MAX_ values of 6.3, 4.8, and 4.3 eV are calculated for X = C, Si, and Ge, respectively. This difference is associated with a qualitative difference in the nature of the molecule orbitals for the carbon QDs as illustrated in Figure 3, which shows the molecular orbitals involved in the Δ*E*_MAX_ transitions. For all QDs, the electron is being excited from an orbital associated with the σ bonding framework. The difference is observed for the orbital receiving the electron, which in the case of C_84_H_86_ has the character of a Rydberg π orbital, whereas for the silicon and germanium QDs, it has a σ/σ*-type character.

**Table 2:** HOMO–LUMO Gap (Δ*E*_HL_, eV), the Excitation Energy for the Lowest Singlet State (Δ*E*_S1_, eV), and Excitation Energy for the Most Intense Transition (Δ E_MAX_, eV) Calculated Using TDDFT CAM-B3LYP with the SRLC Basis Set for Si- and Ge-Based QDs and the 6-31+G* Basis Set for C-Containing QDs

QD	Δ*E*_HL_ (eV)	Δ*E*_S1_ (eV)	Δ*E*_MAX_ (eV)
C_35_H_36_	8.4	6.4	7.2
C_84_H_64_	7.6	6.0	6.3
C_165_H_100_	7.2	5.8	
C_286_H_144_			
C_100_H_60_	7.8	6.2	6.3
Si_35_H_36_	7.7	5.5	5.6
Si_84_H_64_	6.6	4.7	4.8
Si_165_H_100_	6.0	4.3	4.5
Si_286_H_144_	5.5	4.0	
Si_100_H_60_	5.9	4.1	5.0
Si_280_H_120_	5.1	3.6	
Ge_35_H_36_	7.0	4.8	5.0
Ge_84_H_64_	6.0	4.1	4.3
Ge_165_H_100_	5.2	3.7	3.8
Ge_286_H_144_	4.7	3.3	
Ge_100_H_60_	5.0	3.4	4.4
Ge_280_H_120_	4.4	2.9	

Similar behavior is also observed for the HOMO–LUMO gap; the values for the HOMO–LUMO gap presented here are significantly larger than those from previous works.^58,69−71^ This reflects the nature of the CAM-B3LYP exchange-correlation functional compared to the B3LYP functional and other theoretical approaches.

QDs can be compared with a particle in the box problem, and the band gap of the QD can be related to the simple expression for the energy difference between levels for a particle in the box. This suggests that as the size *L* of the semiconductor QD goes up the corresponding energy difference between levels decreases to 1/*L*^2^. The range of QD sizes considered in our manuscript (1–3 nm) is too narrow for a meaningful analogy to be drawn. Takai et al.^72^ provided a simple phenomenological expression applicable to the estimation of the energy gaps at any given temperature in the 0–600 K range, for a wide variety of QD sizes (from very small QDs to bulk).

The electronic structure of the octahedral diamond-like Si and Ge QDs converges toward the corresponding cubic bulk materials as their structure can be superimposed with cubic bulk lattice (octahedral QDs can be considered as finite hydrogenated clusters cut from cubic bulk lattices of silicon and germanium). However, the icosahedral shapes possess slightly different electronic characteristics. Because the icosahedral QD structures are not superimposable with the cubic bulk lattice of Si or Ge, their structures can be considered to approach icosahedral quasicrystals.^73^ This suggests that the electronic characteristics of the icosahedral QDs could be different from the cubic bulk material, as suggested previously for icosahedral silicon quantum dots.^74^ For carbon QDs, the band gap values converge toward that of graphite.

The results show that the HOMO–LUMO gap provides a relatively poor estimate of the excitation energy for the lowest-energy state, indicating that going beyond Kohn–Sham DFT-based molecular orbital picture may be necessary for a reliable description of the photophysical mechanisms. Since the QDs will fluoresce from the S_1_ state, the results show that a wide range of fluorescent energies is possible by tuning the size and composition of the QD.

### 3.2 Dopamine Attachment to QDs

The geometry optimization of smaller, dopamine-functionalized X_35_H_36_ QDs was initially tested using B3LYP DFT and a range of basis sets including 6-31G, 6-31G*, and def2-SVP (see Table S4 in the Supporting Information). The calculated energy values are shown to be not sensitive to the choice of the basis set, and def2-SVP is used in the subsequent analysis of the bigger QDs to obtain the binding energy corresponding to the attachment of dopamine at different sites on the QD surface. This computational setup has been used previously^58^ to study the structure and electronic properties of hydrogenated carbon, silicon, and germanium clusters.

**Figure 4:**
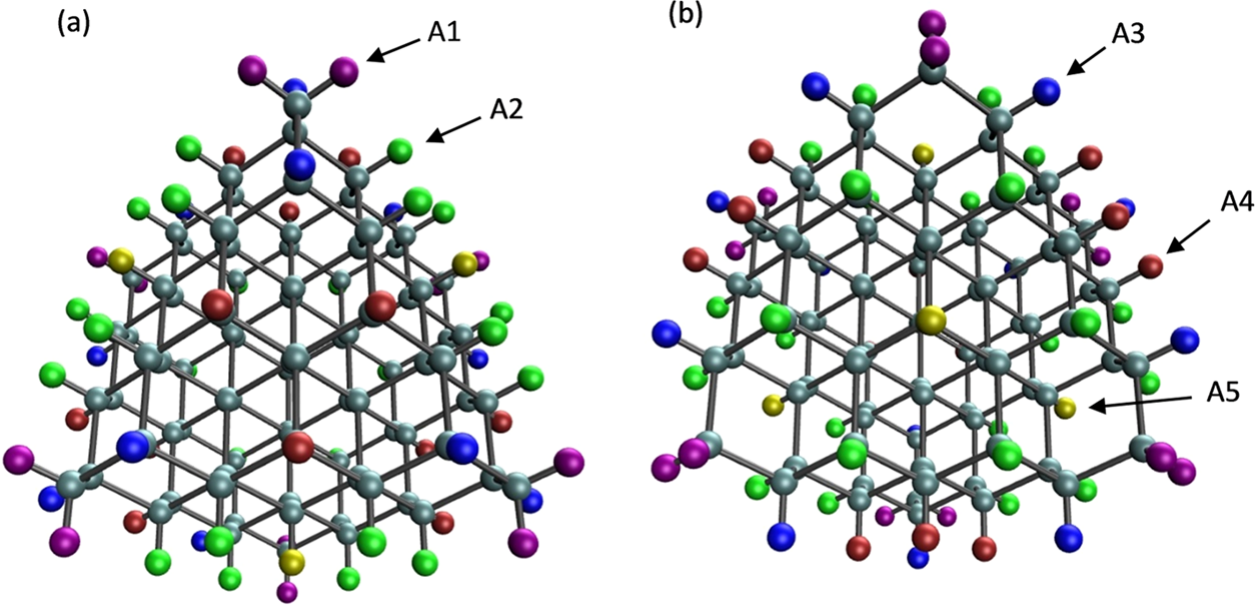
X_84_H_64_ with atoms colored based on their symmetry-equivalent groups.

Figure 4 gives a schematic representation of a diamond-like X_84_H_64_ QD structure, in two different orientations as shown in Figure 4a,b, where terminating hydrogen atoms are colored to distinguish symmetry-equivalent groups. Five distinct adsorption sites (A1–A5) on the surface of Ge_84_H_64_ QD were compared to confirm that the corresponding binding energies are the same for all five sites (see Table S5 in the Supporting Information). This conclusion might have important implications for experimental realization of a QD-based fluorescent sensor, giving a flexibility in its design where the dopamine molecule does not need to be attached to a specific binding site. Note that in QDs with icosahedral structure (Figure 1) all adsorption sites are identical.

### 3.3 Design of a QD for Dopamine Sensing

**Figure 5:**
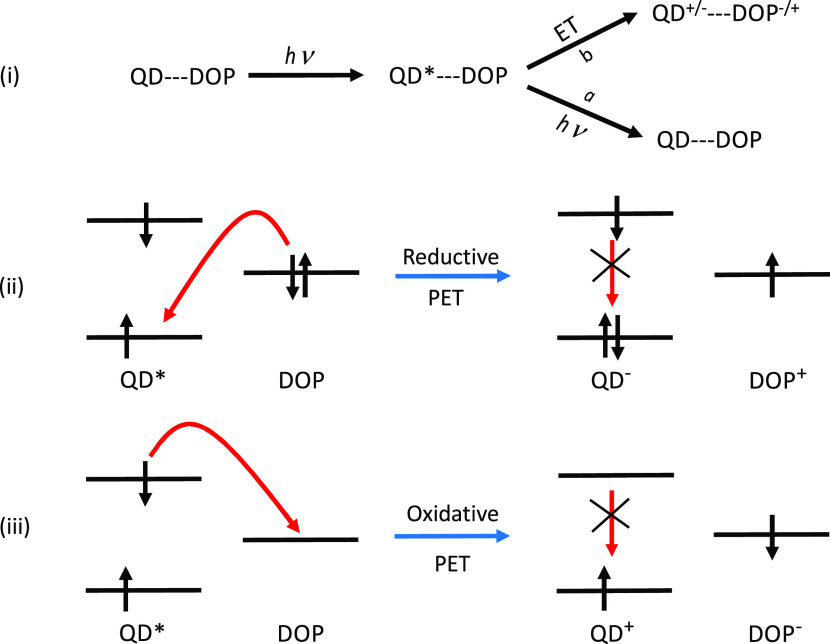
Schematic of the PET mechanism (i), with an illustration of the electronic transitions for (ii) reductive PET and (iii) oxidative PET.

Based upon the electronic structure calculations characterizing the QDs and dopamine, we now explore whether these QDs are suitable to act as the fluorophore in a QD-based fluorescent probe that operates using a PET mechanism. A schematic of this mechanism is shown in Figure 5, which also illustrates how both oxidative and reductive PET mechanisms can occur. The QD undergoes an initial excitation that can lead directly or indirectly via nonradiative processes to the S_1_ state of the QD from which fluorescence can occur, as denoted by path *a*. However, several other photophysical processes may be possible. For example, if dopamine has occupied orbitals that lie higher in energy than the singly occupied orbital of the QD, an electron can transfer from dopamine to the QD in a reductive PET process (the QD is reduced), which has the effect of quenching the fluorescence. Alternatively, if dopamine has unoccupied orbitals lower in energy than the singly occupied orbital of the QD, electron transfer from the QD to dopamine can occur in oxidative PET, which again leads to a quenching of the fluorescence.

In the design of a QD sensor, there are several factors to consider. First, it is desirable for the absorption and emission bands of the QD not to overlap with the transitions of dopamine. The spectra in Figure 2 show that the region 3.75–4.5 eV is suitable, and the calculated transition energies shown earlier indicate that the Si_165_H_100_ and Ge_84_H_64_ QDs would satisfy this condition, while none of the carbon-based QDs studied are suitable. Second, it is required that the PET mechanism occurs leading to quenching of the fluorescence for one form of dopamine and not the other.

**Figure 6:**
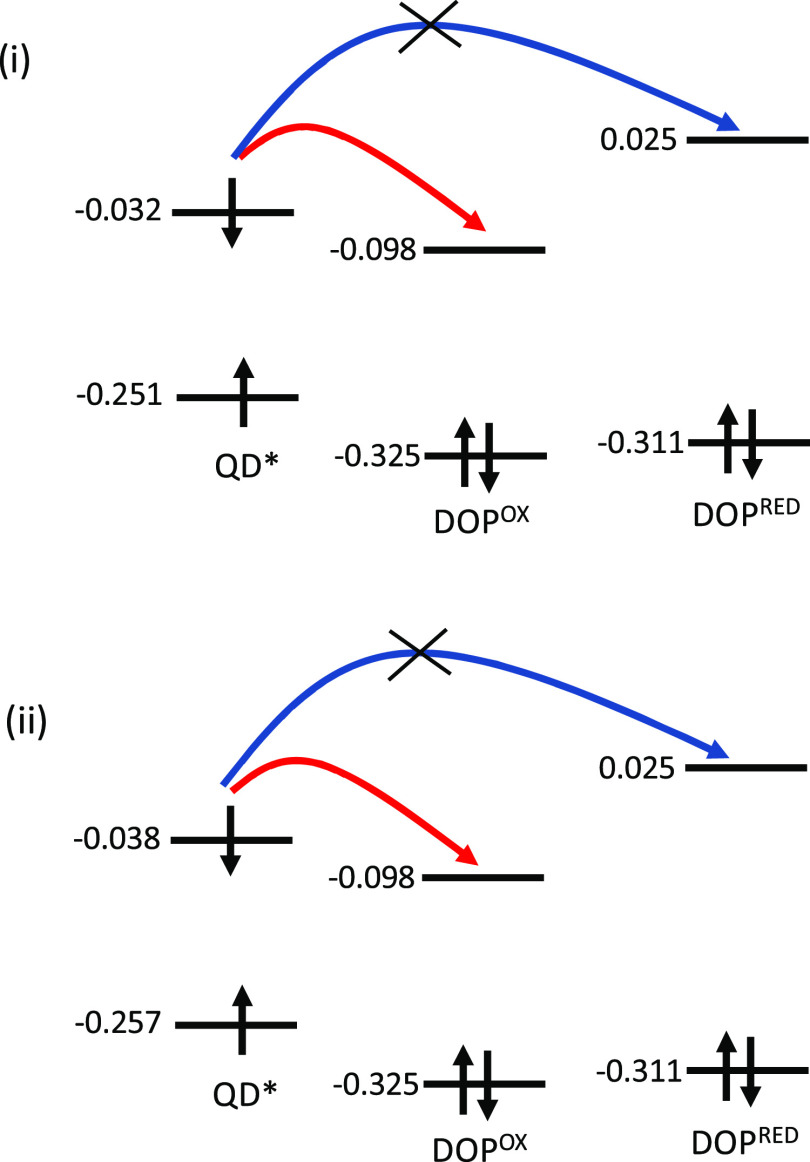
Molecular orbital diagrams showing the HOMO and LUMO energies (in Hartree) based upon the ground-state CAM-B3LYP/SRLC calculation for the Ge_84_H_64_ and Si_165_H_100_ QDs and the reduced and oxidized forms of dopamine.

Initially, it is convenient to examine the molecular orbital diagrams. Figure 6 illustrates the energy difference between the molecular orbitals of Ge_84_H_64_ and Si_165_H_100_ QDs and the reduced and oxidized forms of dopamine. Analysis of these energies suggests that for Ge_84_H_64_ and Si_165_H_100_ QDs oxidative PET would occur for the oxidized form of dopamine but not the reduced form. Consequently, quenching of the fluorescence for the oxidized form of dopamine would be predicted, while the reduced form would continue to fluoresce. This would be consistent with the experimental operation of the biosensor in the original work.^22^ These two QDs are not the only two QDs that fulfill these criteria; others that this level of analysis would predict to be suitable fluorophores include Si_100_H_60_ and Ge_100_H_60_.

**Figure 7:**
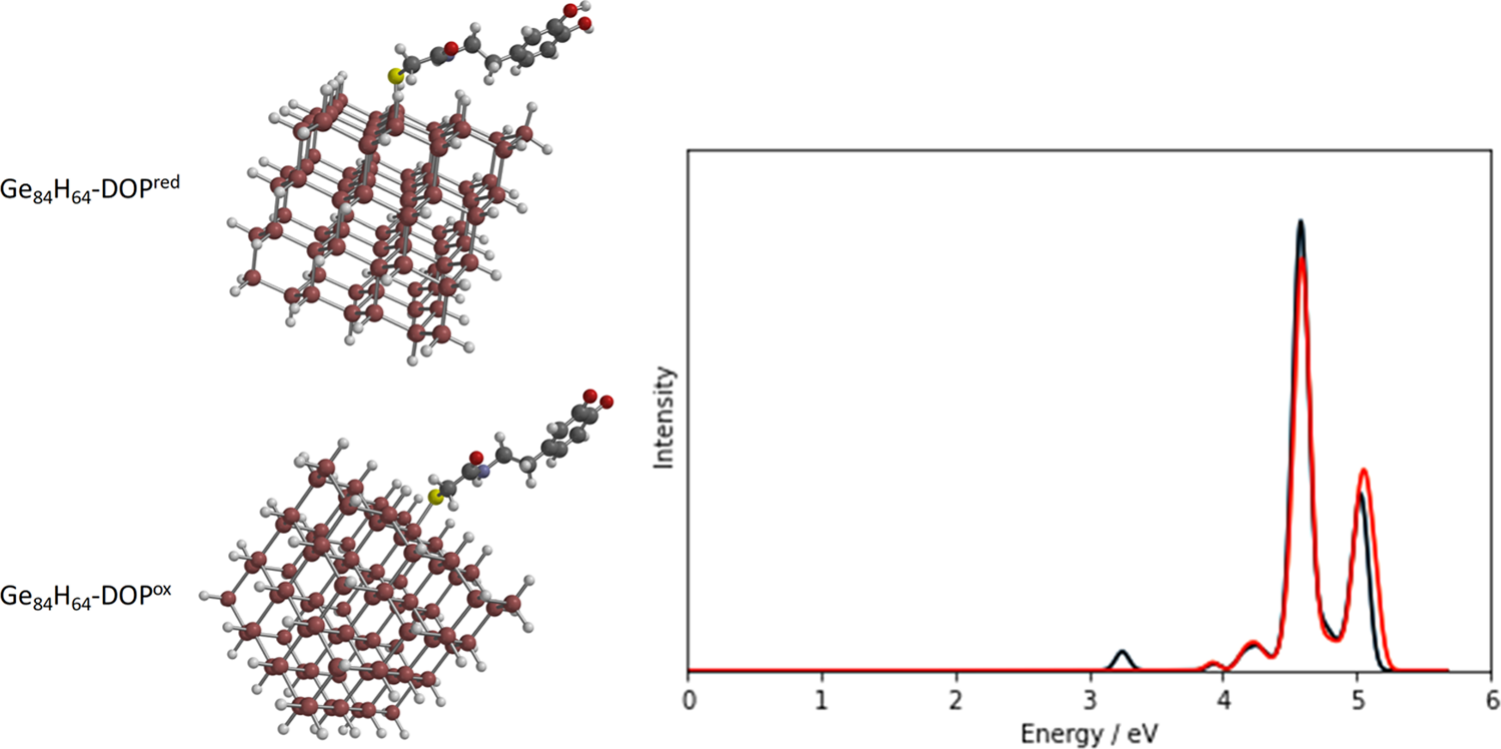
Optimized structures of the two forms of dopamine on Ge_84_H_64_ QD and computed CAM-B3LYP/SRLC spectra for Ge_84_H_64_-DOP^red^ (red line) and Ge_84_H_64_-DOP^ox^ (black line).

Analysis of the photophysical behavior based purely on an orbital energy analysis can be unreliable since differences in orbital energies can provide a poor estimate of the relative energies of the excited states. Going beyond this level of analysis requires explicit consideration of the excited states of the combined QD–dopamine system. Figure 7 shows optimized structures for the oxidized and reduced forms of dopamine on the Ge_84_H_86_ QD (Ge_84_H_86_-DOP^ox^ and Ge_84_H_86_-DOP^red^) along with the calculated absorption spectrum. A similar analysis is included for the Si_165_H_100_ QD in the Supporting Information (Figure S2 and Table S6). The spectra are dominated by the transitions localized on the QD (QD → QD transitions); the exception is the peak at about 3.25 eV for the oxidized form, which corresponds to a transition localized on dopamine (DOP → DOP transition). The absorption spectra can be viewed largely as a superposition of the spectra for the individual dopamine and QD components (see Figure S3 in the Supporting Information) reflecting the relatively small interaction between them. However, the absorption spectra do not reveal the CT transitions that underpin the sensing mechanism since these will have low oscillator strength.

Table 3 shows the low-energy transitions along with a characterization of the nature of the transition. The three-dimensional (3D) visualization of the ground-state and excited-state molecular orbitals contributing to different optical transitions are shown in the Supporting Information (Tables S7 and S8). For the reduced form of dopamine, all of the low-energy states correspond to QD → QD transitions indicating a lack of possibility of forming a state where an electron has been transferred from QD to dopamine that will have lower energy than the S_1_ state formed through excitation on the QD. However, for the oxidized form, there are several QD → DOP transitions that lie at lower energy than the QD → QD transitions; the final state arising from these transitions is consistent with the final state that would arise from the oxidative PET mechanism. These results are consistent with molecular orbital energy-based analysis. This approach to determine the occurrence of PET is based purely on energetic considerations. There are other factors that will determine the efficiency of these processes occurring, such as the rates of surface crossing. While it is possible to take these factors into consideration,^75^ such calculations are much more computationally demanding to be applied on a routine basis.

**Table 3:** Calculated at the CAM-B3LYP/SRLC Level of Theory Transition Energies and Assignments for the Dopamine-Functionalized Ge_84_H_64_ QD

	Δ*E* (eV)	nature of the transition	oscillator strength
Ge_84_H_86_-DOP^red^	3.93	QD → QD	0.0186
	4.13	QD → QD	0.0114
	4.13	QD → QD	0.0006
	4.15	QD → QD	0.0001
	4.17	QD → QD	0.0020
	4.18	QD → QD	0.0035
	4.18	QD → QD	0.0250
	4.20	QD → QD	0.0010
	4.24	QD → QD	0.0020
	4.25	QD → QD	0.0175
Ge_84_H_86_-DOP^ox^	2.12	DOP → DOP	0.0000
	3.24	DOP → DOP	0.0118
	3.25	DOP → DOP	0.0320
	3.67	QD → DOP	0.0001
	3.71	QD → DOP	0.0002
	3.73	QD → DOP	0.0005
	3.84	QD → DOP	0.0000
	3.84	QD → DOP	0.0000
	3.86	QD → DOP	0.0000
	3.94	QD → QD	0.0156

The analysis presented has been based upon gas-phase calculations with no account of solvent. The inclusion of solvent has been shown to be important, particularly for sensing metal ions.^44^ PET leads to the formation of QD^+/–^-DOP^–/+^ species, which will interact more strongly with a polar solvent than the states formed from local QD → QD or DOP → DOP transitions. Consequently, the states formed from QD → DOP transitions are likely to become relatively lower in energy. Table 4 shows the excitation energies and character of the transitions for the Ge_84_H_86_-DOP system calculated using a polarized continuum solvent model (PCM) with a dielectric constant of 80 corresponding to bulk water. For the oxidized form of dopamine (Ge_84_H_86_-DOP^ox^), there is clear evidence that the energies of states formed from QD → DOP transitions have been lowered in energy and these transitions dominate the lowest-energy states except for the lowest-energy DOP → DOP transition. For the reduced form (Ge_84_H_86_-DOP^red^), the introduction of solvent does not have a significant effect on the low-energy states, which are dominated by QD → QD transitions, and the transition energies for these states show a very small change with the introduction of solvent. Overall, the predicted function of the QD in a sensor is not altered, and the presence of additional CT-type transitions for the oxidized form would enhance its effectiveness.

**Table 4:** Calculated at the CAM-B3LYP/SRLC Level of Theory Transition Energies and Assignments for the Dopamine-Functionalized Ge_84_H_64_ QD with the PCM Solvent Model

	Δ*E* (eV)	nature of the transition	oscillator strength
Ge_84_H_86_-DOP^red^	3.92	QD → QD	0.0400
	4.09	QD → QD	0.0312
	4.13	QD → QD	0.0023
	4.14	QD → QD	0.0000
	4.16	QD → QD	0.0090
	4.17	QD → QD	0.0030
	4.17	QD → QD	0.0053
	4.18	QD → QD	0.0011
	4.19	QD → QD	0.0008
	4.20	QD → QD	0.0022
Ge_84_H_86_-DOP^ox^	2.34	DOP → DOP	0.0001
	2.84	QD → DOP	0.0402
	3.16	QD → DOP	0.0001
	3.20	QD → DOP	0.0002
	3.22	QD → DOP	0.0005
	3.31	QD → DOP	0.0000
	3.32	QD → DOP	0.0000
	3.34	QD → DOP	0.0000
	3.51	QD → DOP	0.0004
	3.56	QD → DOP	0.0000

## 4 Conclusions

The use of DFT-based calculations to characterize QDs and to select suitable QDs for a sensing application has been explored within the context of a sensor to differentiate between two forms of dopamine. TDDFT calculations of hydrogenated carbon, silicon, and germanium QDs show that the band gap and fluorescence energies of these QDs vary with size and composition allowing for the possibility of tuning the QDs for a specific application. Additionally, it is shown that the lowest-energy excited singlet states are significantly lower in energy for the oxidized form compared with the reduced form of dopamine. This factor is significant when considering the design of a fluorescence dopamine-functionalized QD sensor. Through an analysis of orbital diagrams and calculations of the excited states of the combined QD-DOP system, it is demonstrated that a sensor could operate based upon a PET mechanism that would occur for the oxidized form of dopamine and not the reduced form. This is evident from low-energy states arising from QD → dopamine transitions that are present for the oxidized form of dopamine. This leads to a quenching of the fluorescence for the oxidized form, allowing the two forms to be distinguished. It is shown that Ge_84_H_64_ and Si_165_H_100_ QDs satisfy the criteria that would make them suitable candidates for the fluorophore in a sensor. Overall, the study highlights how quantum chemical calculations can be used to inform the photophysical processes underpinning the operation of fluorescent probes and presents an approach to support the design of QD-based optical sensors for the detection of biomolecules.

## Supporting Information Available

Computed lowest four singlet transition energies (Δ*E*) in eV for C_35_H_36_, Si_35_H_36_, and Ge_35_H_36_ for a range of computational methods, using TDA approach; calculated transition energies for the lowest four singlet states for functionalized X_35_H_36_ QDs with attached oxidized and reduced forms of dopamine for a range of computational methods; absorption spectra for the oxidized and reduced forms of dopamine with and without the linking group; calculated transition energies for the lowest 10 singlet states for oxidized and reduced forms of dopamine with and without the linking group using TDDFT and TDDFT/TDA methods (CAM-B3LYP/SRLC); calculated energies for functionalized X_35_H_36_ QDs with attached oxidized and reduced forms of dopamine for a range of computational methods; binding energies in a dopamine-functionalized Ge_84_H_64_ QD, calculated at the B3LYP/def2-SVP level of theory; optimized structures of the two forms of dopamine on Si_165_H_100_ QD and computed CAM-B3LYP/SRLC spectra for Si_165_H_100_-DOP^red^ and Si_165_H_100_-DOP^ox^; calculated transition energies and assignments for the dopamine-functionalized Si_165_H_100_ QD; visualized molecular orbitals (with the highest amplitudes) involved in transition for functionalized Ge_84_H_86_-DOP in vacuum and in solvent (PDF)
