# Supplementary material for: Quantum Chemical Characterization and Design of Quantum Dots for Sensing Applications
Source: J Phys Chem A. 2022 May 3;126(19):2899–908. doi: 10.1021/acs.jpca.2c00947 (PMC9125561; doi:10.1021/acs.jpca.2c00947)
Supplement: Supplementary file 1 — jp2c00947_si_001.pdf [file jp2c00947_si_001.pdf]

SUPPORTING INFORMATION

Quantum Chemical Characterisation and Design of Quantum Dots for Sensing Applications

Aleksandra Foerster* and Nicholas A. Besley*

School of Chemistry, University of Nottingham, University Park, Nottingham NG7 2RD, UK

e-mail Aleksandra Foerster: aleksandra.foerster1@nottingham.ac.uk

Table S1 *Computed lowest four singlet transition energies (ΔE) in eV for $C_{35}H_{36}$, $Si_{35}H_{36}$, and $Ge_{35}H_{36}$ for a range of computational methods, using TDA approach.*

ΔE, eV						
	6-31G*	6-31+G*	6-31++G**	def2-SVP	LANL2DZ	SRLC
$Ge_{35}H_{36}$	4.87	4.84	4.84	4.9	ΔE	4.87
	5.03	5.00	5.0	4.9	5.20	5.04
	5.09	5.04	5.03	5.09	5.34	5.14
	5.17	5.12	5.10	5.20	5.41	5.25
$Si_{35}H_{36}$	5.17	5.19	5.18	5.16	5.42	5.54
	5.26	5.20	5.19	5.27	5.68	5.64
	5.28	5.39	5.38	5.3	5.78	5.70
	5.48	5.46	5.45	5.50	5.83	5.87
$C_{35}H_{36}$	8.17	6.45	6.30	7.62	6.08	8.14
	9.18	6.89	6.71	8.43	8.14	9.15
	9.47	7.23	7.06	8.8	9.13	9.49
	9.52	7.29	7.13	8.85	9.47	9.53

Table S2 *Calculated transition energies for the lowest four singlet states for functionalised $X_{35}H_{36}$ QDs with attached oxidised and reduced forms of dopamine for a range of computational methods.*

	SRLC	6-31G*	def2-SVP	6-31+G*
$Ge_{35}H_{36}$+DOP^{ox}	2.11*	2.03*	2.01*	2.1*
	3.22*	3.13*	3.13*	3.16
	3.32	3.40	3.40	3.50
	3.37	3.73*	3.70*	3.50
	3.53	3.94	3.90	3.7
	3.57	4.0*	4.00	3.75*
$C_{35}H_{36}$ + DOP^{ox}	2.12*	2.05*	2.04*	2.07*
	3.14	3.15	3.16*	3.07
	3.24	3.20	3.22	3.15*
	3.51	3.73	3.67	3.54
	3.62	3.85	3.79	3.80
$Ge_{35}H_{36}$+ DOP^{ox}	2.13*	2.04*	2.04*	2.10*
	3.24	3.14*	3.14*	3.16*
	3.28	3.33	3.40	3.48
	4.13	4.37	4.30	3.50
$Ge_{35}H_{36}$+DOP^{red}	4.22	4.27	4.31	-
	4.53	4.59	4.70	-
	4.75	4.80	4.90	-
	4.75	4.81	4.90	-
$C_{35}H_{36}$+DOP^{red}	3.82*	3.87	3.87	3.89
	4.30	4.41	4.39	4.30
	4.76	4.94	4.93	4.90
	5.23	5.18	5.11	5.05
$Si_{35}H_{36}$+ DOP^{red}	4.82	4.90	4.89	4.84
	5.00	5.11	4.94	4.84
	5.20	5.12	5.11	5.04*
	5.31	5.13	5.12*	5.04*

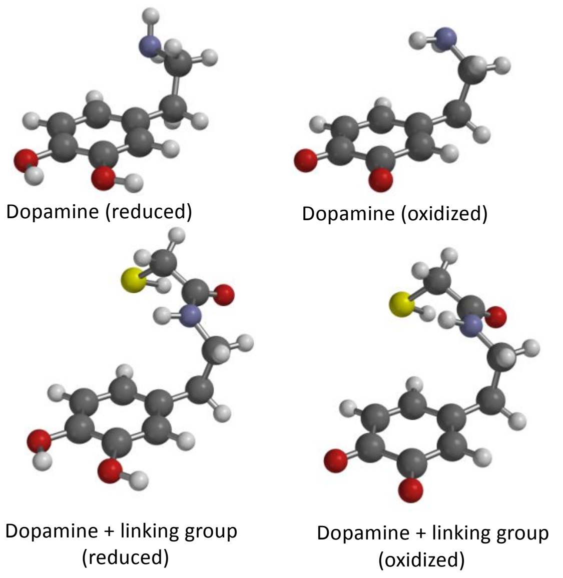

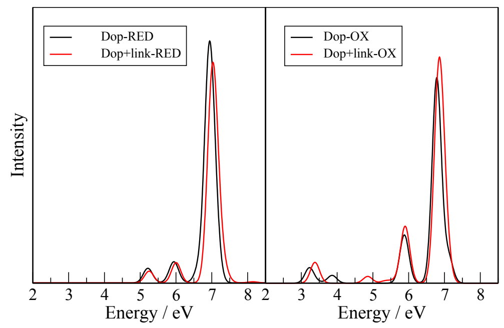

Figure S1: *The oxidised and reduced forms of dopamine with and without the linking group and corresponding absorption spectra.*

Table S3: *Calculated transition energies for the lowest 10 singlet states for oxidised and reduced forms of dopamine with and without the linking group using TDDFT and TDDFT/TDA methods (CAM-B3LYP/SRLC).*

	TDDFT	TDA		TDDFT	TDA
dopamine^{OX}	2.17	2.20	dopamine+link^{OX}	2.17	2.20
	3.23	3.78		3.38	3.55
	3.53	3.57		3.53	3.62
	3.86	3.95		4.63	4.63
	5.88	5.96		4.86	4.87
	5.96	5.98		5.34	5.36
	6.29	6.29		5.56	5.56
	6.42	6.42		5.86	5.88
	6.58	6.59		5.90	5.98
	6.75	6.86		5.99	6.02

	TDDFT	TDA		TDDFT	TDA
dopamine^{RED}	5.24	5.34	dopamine+link^{RED}	5.30	5.20
	6.00	6.06		5.38	5.35
	6.06	6.24		5.87	5.85
	6.84	6.84		6.17	6.23
	6.92	6.97		6.23	6.63
	6.99	7.16		6.67	6.73
	7.09	7.19		6.74	6.90
	7.15	7.36		6.98	6.96
	7.26	7.54		7.04	7.00
	7.43	7.60		7.11	7.04

Table S4 *Calculated energies for functionalised $X_{35}H_{36}$ QDs with attached oxidised and reduced forms of dopamine for a range of computational methods.*

	6-31G	6-31G*	def2-SVP
$Si_{35}H_{36}$-DOP^{ox}	-11218.5634	-11219.5486	-11216.3467
$C_{35}H_{36}$-DOP^{ox}	-2459.2719	-2459.8553	-2458.2843
$Ge_{35}H_{36}$-DOP^{ox}	-73771.9074	-73774.6624	-73774.6957
$Si_{35}H_{36}$-DOP^{red}	-11219.8115	-11220.7887	-11217.5976
$C_{35}H_{36}$-DOP^{red}	-2460.5176	-2461.0932	-2459.5345
$Ge_{35}H_{36}$-DOP^{red}	-73773.1590	-73775.9054	-73775.9500

Table S5 *Binding energies in a dopamine functionalised $Ge_{84}H_{64}$ QD, calculated at B3LYP/def2-SVP level of theory*

H atom replaced with DOP	QD+DOP^{ox}	QD+DOP^{red}
A1	-175555.9062	-175557.1558
A2	-175555.9119	-175557.1528
A3	-175555.9039	-175557.1517
A4	-175555.9067	-175557.1584
A5	-175555.9035	-175557.1498

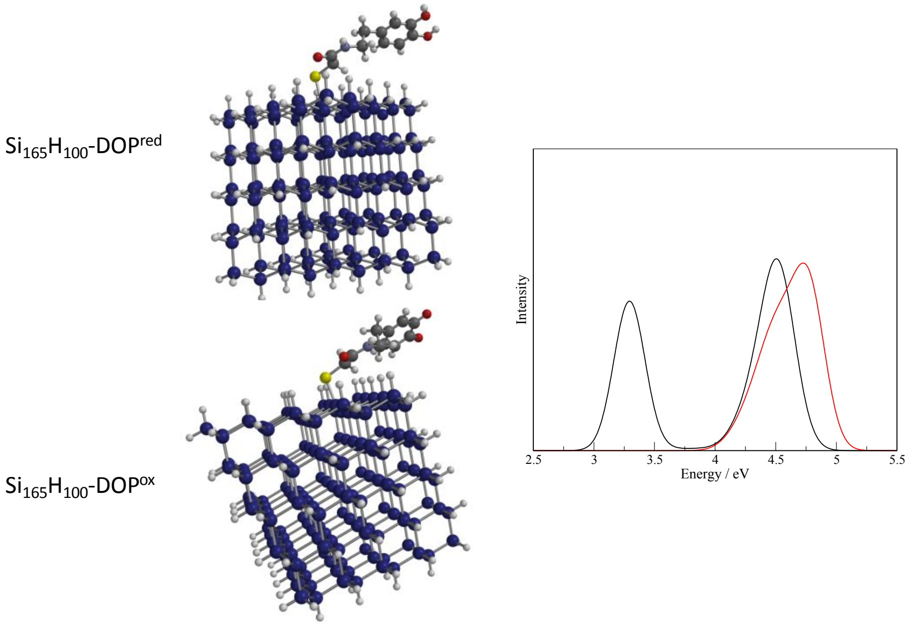

Figure S2: *Optimised structures of the two forms of dopamine on $Si_{165}H_{100}$ QD and computed CAM-B3LYP/SRLC spectra for $Si_{165}H_{100}$-DOP^{red} (red line) and $Si_{165}H_{100}$-DOP^{ox} (black line).*

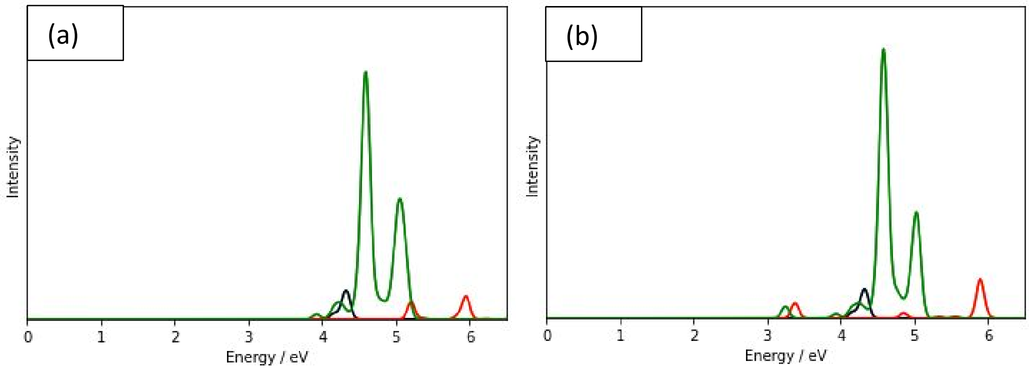

Figure S3 *Computed CAM-B3LYP/SRLC spectra for (a): $Ge_{84}H_{86}$ (black line), DOP^{red} (red line) and $Ge_{84}H_{86}$-DOP^{red} (green line); and (b) : $Ge_{84}H_{86}$ (black line), DOP^{ox} (red line) and $Ge_{84}H_{86}$-DOP^{ox} (green line)*

Table S6: *Calculated transition energies and assignments for the dopamine functionalised $Si_{165}H_{100}$ QD.*

	DE / eV	Nature of the transition
$Si_{165}H_{100}$-DOP^{red}	4.18	QD → QD
	4.25	QD → QD
	4.25	QD → QD
	4.26	QD → QD
	4.26	QD → QD
	4.27	QD → QD
	4.28	QD → QD
	4.43	QD → QD
	4.33	QD → QD
	4.33	QD → QD
	4.34	QD → QD
$Si_{165}H_{100}$-DOP^{ox}	2.13	DOP → DOP
	3.24	DOP → DOP
	3.29	DOP → DOP
	3.30	QD → DOP
	3.34	QD → DOP
	3.37	QD → DOP
	3.37	QD → DOP
	3.52	QD → DOP
	3.53	QD → DOP
	3.62	QD → DOP

Table S7 *Visualised molecular orbitals (with the highest amplitudes) involved in transition (number 1, 4, and 10 excited states) for functionalised $Ge_{84}H_{86}$-DOP in vacuum.*

ΔE / eV	Transition	Occupied orbitals involved in transition	Virtual orbitals involved in transition
2.12	DOP → DOP^{ox}	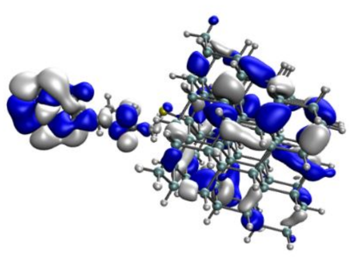	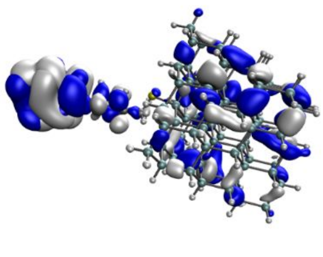
3.67	QD → DOP^{ox}	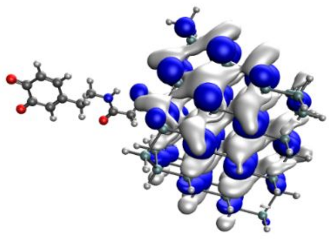	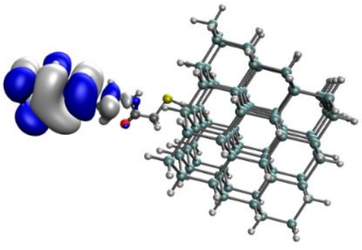

3.94	QD → QD<br>for $Ge_{84}H_{86}$ - DOP^{ox}	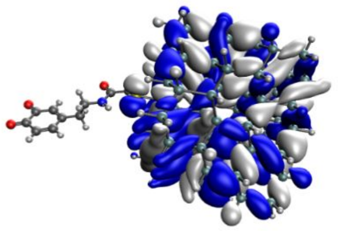	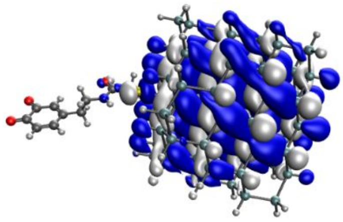
3.93	QD → QD<br>for $Ge_{84}H_{86}$ -DOP^{red}	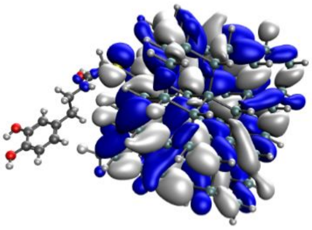	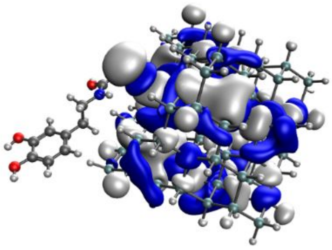

Table S8 *Visualised molecular orbitals (with the highest amplitudes) involved in transition (number 1, 4, and 10 excited states) for functionalised $Ge_{84}H_{86}$ in solvent.*

ΔE / eV	Transiti on	Occupied orbitals involved in transition	Virtual orbitals involved in transition
2.34	DOP^{ox} → DOP^{ox}	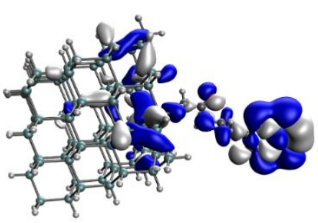	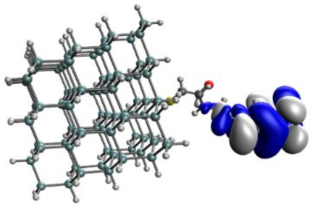
3.20	QD → DOP^{ox}	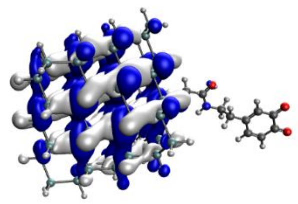	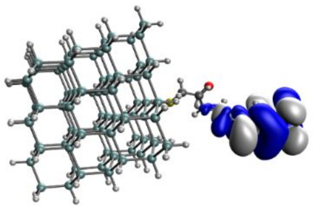
3.92	QD → QD<br>for $Ge_{84}H_{86}$ -DOP^{red}	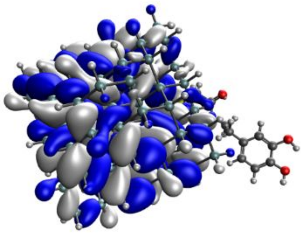	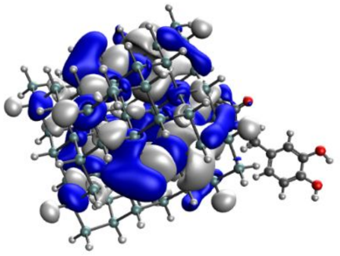

B3LYP-optimised cartesian coordinates of $Ge_{84}H_{86}$-DOP^{ox}, $Ge_{84}H_{86}$-DOP^{red}, $Si_{165}H_{100}$-DOP^{ox}, and $Si_{165}H_{100}$-DOP^{red}

$Ge_{84}H_{86}$-DOP^{ox}

Ge	-0.0511793077	1.3215655550	-2.0860964219
Ge	0.8688013869	-0.9781278184	-2.2566940171
Ge	0.4667061930	-2.2080990251	-0.1443987013
Ge	1.0360196323	2.5089700322	-0.1955986701
Ge	0.6137103605	1.2806256109	1.9144433804
Ge	-2.4971676837	1.1802126991	-1.6813387311
Ge	-1.8350860137	1.1377140624	2.3159078411
Ge	-1.9803692219	-2.3461481033	0.2603357667
Ge	-2.9192853804	-0.0524467937	0.4273694453
Ge	1.5553977521	-1.0113878403	1.7399549112
Ge	-3.4427788525	3.4730826393	-1.5233569015
Ge	-3.5855099278	-0.0041991764	-3.5733043915
Ge	-5.3689236047	-0.1941528190	0.8325086378
Ge	-2.7783645280	3.4302527969	2.4937660263
Ge	-2.2626759046	-0.0866604917	4.4300477468
Ge	-2.4091252580	-3.5865280998	2.3637494934
Ge	-3.0669957605	-3.5442769628	-1.6233626586
Ge	1.1387588308	-2.2487155366	3.8523263058
Ge	4.0194322389	-0.8820872778	1.3536996118
Ge	1.6914295767	2.4720864088	3.8090465665
Ge	1.4061381213	-4.5048976066	-0.3112424252
Ge	3.4898683429	2.6760249871	-0.6031278311
Ge	0.1022744264	4.8080585005	-0.0305197071
Ge	3.3044364692	-0.8508299720	-2.6905475815
Ge	-0.1972479299	-2.1718730341	-4.1530504637
Ge	0.3745226536	2.5542665353	-4.1974293753
Ge	4.3322005536	0.3470373187	-0.7832173438

Ge	-1.3097570917	-2.3620282739	4.2100000251
Ge	-2.6279379400	-2.2844080702	-3.7041732492
Ge	-2.3343843480	4.6184190031	0.3692876345
Ge	5.0442809355	0.3059707677	3.2769054055
Ge	4.9218284437	-3.1852851127	1.1863353083
Ge	3.8423075308	-4.3325245372	-0.7169344216
Ge	0.9737896907	-5.7103533610	1.8049676150
Ge	2.0945280355	-4.5275976128	3.6613169591
Ge	2.2177944809	-1.0465833259	5.7274741112
Ge	1.2533642838	1.2220705671	5.8965428998
Ge	4.1272301366	2.6013419034	3.3850545802
Ge	4.5282537903	3.8729461985	1.3050183053
Ge	3.8837642199	3.9053189704	-2.7133656291
Ge	3.7234934841	0.3894824300	-4.7890436380
Ge	4.2392903371	-3.1398490506	-2.8409849773
Ge	0.2488456269	-0.9226543363	-6.2404764016
Ge	0.7692666892	-4.4477536068	-4.2977689899
Ge	2.8181611269	2.6768247532	-4.5726220376
Ge	-0.5712661234	4.8346987341	-4.0014009101
Ge	-0.7186122320	1.3447373515	-6.0592664128
Ge	0.5461505806	6.0181108382	-2.1436123285
Ge	1.1912581603	5.9762622758	1.8597377227
Ge	-3.0116149898	4.6778382957	-3.6435875275
Ge	-5.8831398198	3.3018439215	-1.1266240103
Ge	-6.0228546380	-0.1234313339	-3.1484162767
Ge	-3.1560582328	1.2514332615	-5.6613762454
Ge	-6.2786004682	2.1033175607	0.9960063389
Ge	-6.4231031887	-1.3850427066	-1.0641676125
Ge	-5.7608330567	-1.4252288264	2.9399406300
Ge	-5.2169309093	3.2602711959	2.9028723176
Ge	-1.6904823766	4.5907557853	4.3915854533

Ge	0.7358907843	4.7506912486	3.9534691220
Ge	-1.1823131795	1.1260792386	6.2989038292
Ge	-4.7080089231	-0.2035105108	4.8109476531
Ge	-1.4659563891	-5.8700546618	2.1682586931
Ge	-4.8550374171	-3.7159492195	2.7374092985
Ge	-5.5027804563	-3.6760589382	-1.1927985485
Ge	-2.1180079115	-5.8283802356	-1.7626252997
Ge	0.3095182556	-5.6673474558	-2.2000477645
Ge	4.6591780707	-0.9575030742	5.3645744576
Ge	4.5411879087	-4.3861880281	3.3158590074
Ge	-5.6358637726	2.0799211877	5.0355909165
Ge	-2.1583054488	3.3898487599	6.5026337714
Ge	-6.9687720856	2.1614714891	-3.0349046554
Ge	-4.1400771227	3.5178323749	-5.5148547670
Ge	-5.9256550988	-4.9521258650	0.8810354584
Ge	-2.5843518561	-7.0756663744	0.3214878108
Ge	2.9858706648	6.2032434036	-2.5104007610
Ge	3.6183374873	6.1690051268	1.4385722091
Ge	2.6829016271	-0.8321859620	-6.6715141058
Ge	3.1974664578	-4.3083711767	-4.7556575005
Ge	-5.0147588320	-7.2386448909	0.7118370680
Ge	4.0607482676	7.4078398684	-0.6459841792
Ge	-4.5870990684	3.2770835189	6.9188255379
Ge	3.6458942629	-3.0952607057	-6.8545948647
Ge	5.6327251772	-3.2185320658	5.1946251741
Ge	-6.5738396541	3.4013725383	-5.1296115370
H	1.4353903991	5.4780490201	5.1442838002
H	-1.5761954686	-3.1332115987	5.5422647454
H	-2.9146548757	6.0643299029	0.4859401704
H	-3.3064016184	-3.0440150168	-4.8889265337
H	1.8884244672	-1.8348798518	7.0328349158

H	6.5815619624	0.3837502530	3.0290717335
H	1.7834404741	-5.2656828972	5.0001120550
H	6.4556960884	-3.0651989495	0.9241455835
H	-4.9308686465	-0.9995779817	6.1341250725
H	-5.7662571338	4.7181875973	2.9936728955
H	-1.4627854646	0.3139024695	7.6011568327
H	-2.2859938768	6.0312666919	4.4620549295
H	-6.4308631189	4.7575893862	-0.9997749694
H	-6.6536368311	-0.9030751748	-4.3438704170
H	-3.5929874997	6.1167221963	-3.4828912785
H	-3.8245223346	0.4557499139	-6.8251414482
H	-6.1358454625	-4.4262597200	-2.4059024217
H	-2.8007613848	-6.5474925673	-2.9676141145
H	-5.0812130277	-4.4750843012	4.0814595892
H	-1.7457580254	-6.5975382258	3.5195460929
H	0.5596358010	7.3991906336	1.9551719499
H	-0.0693403460	7.4435581704	-1.9930751282
H	5.4232540525	3.9696598449	-2.9394570044
H	6.0603364651	3.9629264008	1.0417678160
H	5.2650176438	0.4615216127	-4.9880787313
H	-0.4606454553	-1.6939566744	-7.3963817469
H	5.7742190453	-2.9959420932	-3.0705295040
H	0.0570203069	-5.1803232629	-5.4773119740
H	3.7665595631	-5.7582869600	-4.8233589131
H	2.9262442085	-0.0243070039	-7.9817761170
H	-1.4368800745	4.1381462573	7.6643375428
H	-7.1769350801	1.9584132828	5.2389566153
H	5.1016386382	-5.8334042562	3.1654207073
H	5.3058066923	-0.1585292208	6.5355701009
H	-3.8127108743	4.2771148728	-6.8360242913
H	-8.4963773084	2.0431957581	-2.7477070079

H	4.2732982251	6.8870484344	2.6567706895
H	3.2345545916	6.9425849811	-3.8595840906
H	-7.4634450155	-4.9917522987	1.1290581711
H	-1.9549609862	-8.4957732864	0.1962876001
H	-7.2710016950	2.6839865646	-6.3149316255
H	-7.1543624031	4.8351459882	-5.0085325475
H	7.1599854748	-3.1116412354	4.9420339178
H	5.4076873159	-4.0033936617	6.5131454649
H	5.5894064865	7.5117956414	-0.8853617719
H	3.4793032950	8.8417619068	-0.5341891658
H	-5.6828481446	-7.9972821384	-0.4652126330
H	-5.3016040642	-8.0185419953	2.0218277715
H	-5.1642009100	4.7137443719	7.0210762220
H	-4.8795317280	2.5388027233	8.2517417844
H	5.1780727873	-2.9884872189	-7.0775898754
H	3.0280561511	-3.8567015227	-8.0570527984
H	4.4015942207	-5.7860878995	-0.8284064225
H	0.9167008353	-7.1025802041	-2.2879984382
H	1.5703703494	-7.1448124447	1.6561621096
H	-0.2728800367	5.5739161447	-5.3430314442
H	-0.4180827060	2.1363997781	-7.3703841061
H	3.0511295251	3.4520787180	-5.9066891281
H	-7.3044946262	-1.4993540002	3.1570673117
H	-7.9570323243	-1.4580832269	-0.7871854879
H	-7.8151676460	1.9734331822	1.2406539065
H	4.7697278928	3.3619684345	4.5864951420
H	1.9443247449	2.0005585652	7.0593540343
S	6.5654149470	0.3560626765	-1.4909223337
C	7.4921181610	1.2345151775	-0.0755347315
H	7.0714440642	0.8941172502	0.8743031768
H	7.3428194136	2.3105742484	-0.1578699151

C	8.9919031883	0.9769942664	-0.0754285339
O	9.7569920904	1.7721825153	0.5186118897
N	9.4432249829	-0.1437336604	-0.7062695590
H	8.7739573969	-0.7422708349	-1.1720470779
C	10.8698475844	-0.4465815531	-0.7850456614
H	11.4121072958	0.4825328468	-0.9864806506
H	11.0257290699	-1.1248750266	-1.6299295487
C	11.4218183441	-1.0667391277	0.5287013956
H	10.8388978065	-1.9671916800	0.7641087838
H	11.2624642302	-0.3405393188	1.3303743323
C	12.8868605945	-1.4097682698	0.4085473337
C	15.6438781345	-2.1974098349	-0.0244601340
C	13.8606677303	-0.6111666625	0.9241597259
C	13.2501840663	-2.6276589644	-0.3341983769
C	14.5334123981	-3.0049629388	-0.5414129530
C	15.2800574118	-0.9234153187	0.7608171023
H	13.6211301820	0.2926834245	1.4747544082
H	12.4358796098	-3.2374376381	-0.7170465807
H	14.7939685526	-3.9055336190	-1.0865745909
O	16.1819030028	-0.1996420497	1.2279144826
O	16.8352395446	-2.5142687555	-0.2027841459

$Ge_{84}H_{86}$-DOP^{red}

Ge	-0.1355085723	1.7214208605	-2.1482955998
Ge	0.9339527323	-0.4445247500	-2.7273609295
Ge	0.9250152539	-1.9381918038	-0.7444470833
Ge	1.1018846340	2.7910904940	-0.2803083383
Ge	1.0709165935	1.2945800048	1.7016213197
Ge	-2.4871655294	1.3003532130	-1.4678353979
Ge	-1.2837144123	0.8723507581	2.3786553587

Ge	-1.4275601037	-2.3568203771	-0.0637186354
Ge	-2.5170221714	-0.1995177113	0.5125632110
Ge	2.1719427801	-0.8568875058	1.1143613313
Ge	-3.5844291946	3.4554125639	-0.8958494043
Ge	-3.7223998939	0.2335853564	-3.3392099123
Ge	-4.8708558404	-0.6207417056	1.1935875169
Ge	-2.3785760407	3.0254400302	2.9704895272
Ge	-1.3224883995	-0.6226415078	4.3633182990
Ge	-1.4647376698	-3.8671288751	1.9087958409
Ge	-2.6614633705	-3.4381993530	-1.9290345723
Ge	2.1369400343	-2.3608600036	3.0954316711
Ge	4.5516699358	-0.4437075231	0.4532654666
Ge	2.2857419457	2.3713914001	3.5809071990
Ge	1.9961410014	-4.1050929317	-1.3282234454
Ge	3.4613904772	3.2377711376	-0.9580542084
Ge	0.0125987238	4.9535994489	0.2980754733
Ge	3.2751262455	-0.0387413005	-3.4323625179
Ge	-0.2786071365	-1.5165332837	-4.6081198327
Ge	-0.0994162963	3.2235497976	-4.1294919780
Ge	4.4605965820	1.0330920636	-1.5436883338
Ge	-0.2240268931	-2.7540175183	3.7365781056
Ge	-2.6092987632	-1.9114126969	-3.8788656218
Ge	-2.3247263941	4.4834310812	0.9686522687
Ge	5.7282498539	0.6228272741	2.3563220087
Ge	5.5645962028	-2.6313106150	-0.1250414864
Ge	4.3290755975	-3.6566646843	-1.9975650860
Ge	1.9605402883	-5.5780642285	0.6614925013
Ge	3.2273290076	-4.5044857674	2.4896508829
Ge	3.3562481197	-1.2662005725	4.9510484663
Ge	2.2319983974	0.8537486985	5.5349127114
Ge	4.6230933615	2.7714381795	2.8814287392

Ge	4.6268740500	4.3139437129	0.9482285839
Ge	3.4587737098	4.7311288035	-2.9348832421
Ge	3.3081138371	1.4688981002	-5.3966066356
Ge	4.3527008012	-2.1981310185	-3.9931762387
Ge	-0.2171886570	0.0047181656	-6.5627424924
Ge	0.8358258570	-3.6590795831	-5.1650328324
Ge	2.2555889771	3.6198662518	-4.7827589723
Ge	-1.1888934843	5.3636199251	-3.5251248270
Ge	-1.3326600957	2.1307738636	-5.9742661831
Ge	0.0703412630	6.4340657837	-1.6891397492
Ge	1.2393385136	6.0098267007	2.1706889950
Ge	-3.5380779255	4.9300468689	-2.8889363464
Ge	-5.9280070349	3.0056012188	-0.2283209139
Ge	-6.0634426829	-0.1673446413	-2.6347038662
Ge	-3.6787002133	1.7555620166	-5.2939400310
Ge	-5.9305624558	1.5421972514	1.7641213201
Ge	-6.0720291081	-1.6883011980	-0.6853641345
Ge	-4.8719790538	-2.1180391792	3.1646323993
Ge	-4.7173087601	2.5745820816	3.6498697376
Ge	-1.1488551289	4.0709794628	4.8475430051
Ge	1.1740524105	4.5127610978	4.1357017417
Ge	-0.1099367959	0.4759621108	6.2198499840
Ge	-3.6766653121	-1.0200766434	5.0238928913
Ge	-0.3845496329	-6.0149019274	1.3033711171
Ge	-3.8202211472	-4.2720958229	2.5616198347
Ge	-4.9987681073	-3.8503868628	-1.2175019658
Ge	-1.5670335351	-5.5922102258	-2.4804158865
Ge	0.7558433013	-5.1499098296	-3.1950907654
Ge	5.7132842626	-0.9032415928	4.3029873127
Ge	5.5901513097	-4.0882476244	1.8770999151
Ge	-4.7490593525	1.1208588095	5.6525666945

Ge	-1.2317957581	2.5964987139	6.8325243838
Ge	-7.1638458518	1.9858204915	-2.1135397774
Ge	-4.8113809953	3.8829925932	-4.7343915345
Ge	-5.0329117222	-5.3902754667	0.7190616230
Ge	-1.6453713552	-7.1071170885	-0.5238316348
Ge	2.4154210992	6.8939763019	-2.3301395474
Ge	3.5625836189	6.4819031542	1.4781287500
Ge	2.1242145900	0.3748795098	-7.2697924992
Ge	3.1618200840	-3.2372567274	-5.8938509167
Ge	-3.9690539346	-7.5491140096	0.1765963236
Ge	3.6387436047	7.9770054334	-0.4830568500
Ge	-3.5632874009	2.1935188390	7.5283233872
Ge	3.1970488943	-1.7642665542	-7.8719876368
Ge	6.7440403304	-3.0876093020	3.8168667391
Ge	-7.1545701984	3.4929709192	-4.0674624983
H	1.9746392654	5.1565714329	5.3105396577
H	-0.2335130470	-3.6871311629	4.9897499850
H	-3.0085114889	5.8410226457	1.3291799244
H	-3.3891178921	-2.5839445504	-5.0539002482
H	3.2883407531	-2.2259738256	6.1787094337
H	7.1833338793	0.9328126202	1.9274789861
H	3.1762878638	-5.4099160605	3.7596390401
H	7.0171937021	-2.3893572487	-0.6303929266
H	-3.6544942261	-1.9707535409	6.2608267043
H	-5.3724164117	3.9497022402	3.9876405594
H	-0.1397223425	-0.4954060555	7.4399480771
H	-1.8539438254	5.4256681500	5.1671707896
H	-6.5762385758	4.3758901481	0.1404414697
H	-6.7952050225	-0.8602742756	-3.8262082869
H	-4.2200974986	6.2750186327	-2.4887449731
H	-4.4356579136	1.0398957429	-6.4557555279

H	-5.7451463670	-4.5020041806	-2.4231621720
H	-2.3626019691	-6.2199411290	-3.6668967804
H	-3.8011386862	-5.1914204950	3.8225085025
H	-0.4125940126	-6.9120160604	2.5800317217
H	0.5017849038	7.3407678091	2.5153871562
H	-0.6439124359	7.7642769424	-1.2956532691
H	4.9400836911	4.9660392619	-3.3541803376
H	6.0938790307	4.5885386529	0.5034042917
H	4.7970768720	1.7034929849	-5.7796204092
H	-1.0127954493	-0.6867619241	-7.7131790822
H	5.8259998782	-1.8993521056	-4.3996817612
H	0.0240602555	-4.3052982720	-6.3303741669
H	3.8365678655	-4.6056306489	-6.2129311475
H	2.1214389486	1.3383238410	-8.4948314493
H	-0.4199206317	3.2645207050	7.9830976908
H	-6.2352706915	0.8392502045	6.0264992859
H	6.2575529327	-5.4418890211	1.4837767238
H	6.4456813900	-0.1933399151	5.4807707662
H	-4.7411667321	4.8110385829	-5.9845893689
H	-8.6213423339	1.6965408156	-1.6424716683
H	4.3325180559	7.1054330340	2.6810352277
H	2.4132071815	7.7978594116	-3.6005785321
H	-6.5123473339	-5.5975885198	1.1634777098
H	-0.9187094423	-8.4347486248	-0.8971716427
H	-7.9483017168	2.8638984646	-5.2430421261
H	-7.8255573879	4.8401116199	-3.6901205368
H	8.2624329130	-2.9530647698	3.5693588797
H	6.5188680508	-4.0212009392	5.0366885732
H	5.1108682512	8.2262406084	-0.9027375866
H	2.9836144914	9.3417584218	-0.1441923930
H	-4.7487459486	-8.2705745987	-0.9540255773

H	-3.9737416143	-8.4547765732	1.4368255152
H	-4.2522673163	3.5359437299	7.8902631009
H	-3.5918939456	1.2750894758	8.7787158494
H	4.6592208402	-1.5237732769	-8.3310069984
H	2.4186730708	-2.4305440629	-9.0372100162
H	4.9974009200	-5.0233847287	-2.3474851989
H	1.4690733991	-6.4970366199	-3.5312027534
H	2.6626435279	-6.9152152379	0.2672614535
H	-1.1494398843	6.2710281597	-4.7946745155
H	-1.2899124182	3.0862806059	-7.2073784100
H	2.2419601343	4.5530050619	-6.0340071705
H	-6.3598327586	-2.3574951406	3.5694598237
H	-7.5401294042	-1.9336297406	-0.2155498576
H	-7.4011518625	1.2465049303	2.1943645867
H	5.3663014093	3.4474987023	4.0749788069
H	3.0126158362	1.5511656429	6.6924037532
S	6.5612157502	1.3351551506	-2.5481976519
C	7.6106691848	2.2529034825	-1.2502708523
H	6.9524612733	2.7333262780	-0.5255008867
H	8.1278156694	3.0448772936	-1.7978936859
C	8.6526052669	1.4374976969	-0.4937154404
O	9.3087632013	1.9982962373	0.4183171802
N	8.8429473132	0.1454353892	-0.8629486250
H	8.2401892598	-0.2397014435	-1.5789488099
C	9.8855366035	-0.6887333335	-0.2592143343
H	10.7969981290	-0.0890013585	-0.1674808904
H	10.0897659738	-1.5173925043	-0.9445856883
C	9.4970355664	-1.2411114153	1.1342723937
H	8.5414800195	-1.7689645335	1.0424564285
H	9.3396414844	-0.3879881032	1.8028985915
C	10.5492805148	-2.1737945606	1.6965162674

C	12.5218855680	-3.8989145218	2.6952128212
C	11.6715334604	-1.6564302615	2.3681081993
C	10.4366856428	-3.5644784515	1.5316634961
C	11.4210898125	-4.4291340674	2.0310620146
C	12.6516612419	-2.5106624487	2.8649201438
H	11.7905186766	-0.5890612177	2.5187745874
H	9.5724215065	-3.9771964225	1.0195935622
H	11.3218803976	-5.5041036357	1.9053320929
O	13.7500301417	-1.9926707939	3.5299320597
H	14.3336205479	-2.7265423112	3.8147830040
O	13.5721050944	-4.6460993639	3.2475032151
H	13.4572364607	-5.6047164545	3.1205580736

$Si_{165}H_{100}$-DOP^{ox}

Si	-0.5410122964	-0.0264178776	-0.0388836296
Si	1.6076750304	0.5701988206	-0.9856254670
Si	-2.3059718147	1.0558108172	-1.2896009344
Si	-0.8312751666	-2.4261684293	-0.1489383936
Si	-0.6279146966	0.7020675602	2.2648349493
Si	-2.2153354537	0.3278151877	-3.5937623108
Si	-2.0153307044	3.4548196230	-1.1775072215
Si	-4.4531729626	0.4591214632	-0.3415219617
Si	-2.7761993854	0.1050759619	3.2094471708
Si	-0.3356368570	3.1005013597	2.3737606425
Si	1.8886603723	2.9695216753	-0.8713753692
Si	1.6999158660	-0.1557994040	-3.2897161440
Si	3.3717853044	-0.5092984054	0.2681299767
Si	-0.7414712414	-3.1515337718	-2.4537305921
Si	-2.9795960819	-3.0198838234	0.7977485431
Si	0.9335619426	-3.5055591601	1.1032838192

Si	1.1355975483	-0.3804375292	3.5144578592
Si	1.4070244431	-2.5549157264	-3.3979044826
Si	3.0817475616	-2.9109498333	0.1560480276
Si	0.8437901835	-2.7796084450	3.4067350217
Si	-3.0658772348	-2.2929239926	3.1006949479
Si	-4.7421612938	-1.9393671350	-0.4542837069
Si	-2.5070559203	-2.0706815029	-3.7027032729
Si	-0.0673652111	0.9249530743	-4.5382315587
Si	0.1291489548	4.0508027471	-2.1269097168
Si	1.8124103021	3.6977254771	1.4300495081
Si	-2.1018001433	4.1801634709	1.1257902184
Si	3.2841342850	0.2196734597	2.5700012274
Si	-4.5391385986	1.1874410612	1.9611094778
Si	3.1695344750	-3.6386064988	-2.1468075312
Si	3.5843113768	2.6178644117	2.6699689123
Si	0.2196339345	3.3231138201	-4.4293373396
Si	-4.6545795405	-2.6672264695	-2.7575327060
Si	-1.3041738038	-3.3746830487	4.3520439964
Si	-4.2482581948	3.5849615146	2.0728941261
Si	2.8855544600	-6.0353698382	-2.2599007000
Si	5.3131057447	-3.0415817022	-3.0887425144
Si	1.4933715676	-3.2838097233	-5.6981966761
Si	-1.0283960222	-5.5469021241	-2.5658216813
Si	0.6467846429	-5.9011472363	0.9910975109
Si	4.8346603802	-3.9999176593	1.4108210641
Si	5.5187325555	0.0868047692	-0.6828186546
Si	3.8448277608	0.4434174815	-4.2307426433
Si	0.0185837819	0.1960868241	-6.8380890981
Si	-2.4197071412	-2.7985997946	-6.0032065818
Si	-3.2663029005	-5.4140974992	0.6856699199
Si	-4.9429431340	-5.0625086054	-2.8710793380

Si	-6.4179517433	-1.5870319435	-4.0032819790
Si	-6.8853396438	-2.5331185829	0.4919985149
Si	-5.2095398836	-2.8867191730	4.0453788943
Si	-6.6826785131	0.5931936626	2.9056756799
Si	-6.2159162154	1.5391789814	-1.5857374457
Si	-2.8610444965	0.8334847574	5.5067042296
Si	1.0490572039	0.3464692506	5.8127475716
Si	5.0299893163	-0.8735202237	3.8280652120
Si	5.7401703648	3.2334527759	1.7373150676
Si	3.4888936801	3.3422417049	4.9722556451
Si	-0.4230294409	3.8280156677	4.6708991608
Si	2.1034286773	6.0915198128	1.5397053894
Si	4.0217464719	3.5752524697	-1.8229544463
Si	2.3618723974	3.9194139085	-5.3727824928
Si	-1.5427627472	4.4035597277	-5.6754745869
Si	0.4186374561	6.4451945897	-2.0139005378
Si	-3.7788159767	4.5330261264	-2.4222182386
Si	-1.8133416141	6.5746923687	1.2385044727
Si	-4.3344881927	4.3143207759	4.3710855060
Si	-6.0124307185	4.6660133440	0.8300782976
Si	-3.9793037712	1.4086342349	-4.8377125174
Si	-1.3906493358	-2.6474676901	6.6515350291
Si	-1.5924440949	-5.7702453514	4.2426986268
Si	2.6014090974	-3.8650569700	4.6573303760
Si	5.5737758571	-0.6533341910	-2.9699797904
Si	4.0936149895	2.8321072881	-4.1091978889
Si	5.7581797274	2.4860254839	-0.5581441882
Si	0.3281767848	7.1228077283	0.2888178398
Si	-3.6579690437	3.7887022918	-4.7051648644
Si	-5.8943346446	3.9192762883	-1.4519782495
Si	-6.0953465305	0.7928305560	-3.8684868973

Si	-6.9343666431	-1.7945775019	2.7789138314
Si	-2.5668874242	3.2188950668	5.5756695145
Si	-1.0962738408	-0.2615244979	6.7156049312
Si	1.3438409482	2.7324933904	5.8743106945
Si	4.7161893252	-3.2521355784	3.6915029246
Si	-3.1716804576	-6.0968361490	-1.6166368784
Si	-1.4967254959	-6.4505866259	1.9399297521
Si	0.7418259190	-6.5840141321	-1.3111250033
Si	-0.2743665446	-2.1889961607	-6.9042096449
Si	2.6175425811	6.3072254673	-5.2494444835
Si	-1.3502494613	7.4865741224	-3.2622837693
Si	-1.2284325759	6.7837760844	-5.5477720677
Si	-1.4278617238	3.6600460565	-7.9584310168
Si	2.4188689095	3.1815457222	-7.6597804908
Si	2.1605280335	0.8017885348	-7.7450424260
Si	-1.7463472696	1.2875791759	-8.0482590150
Si	4.3026309209	5.9590722193	-1.6947031485
Si	2.5572311682	7.0035515280	-2.9591139443
Si	4.2430735491	6.6492690201	0.5959897429
Si	3.8983867353	-0.2965599293	-6.5163049340
Si	-3.8666770718	0.6660572337	-7.1203183882
Si	-3.4662192618	6.9124351956	-2.2941522992
Si	-5.6970467004	7.0465343897	0.9601724250
Si	-8.1323563505	4.0559528107	1.7920751776
Si	-8.3353208401	0.9300348339	-0.6249115418
Si	-8.5372963595	-2.1971017815	-3.0400671775
Si	-6.3042978181	-2.3297677947	-6.2871705043
Si	-4.1838880078	-1.7063574405	-7.2135365432
Si	-2.7100750333	-5.1827164866	-6.0810898082
Si	1.2042930688	-5.6683693641	-5.7754275297
Si	3.6357498301	-2.6761028518	-6.6037820103

Si	2.9783592799	-6.7246345259	-4.5605050800
Si	4.6554736062	-7.0778855227	-1.0090780850
Si	-0.9379632226	-6.2393656097	-4.8642911289
Si	-4.8529924502	-5.7523610355	-5.1715219428
Si	-7.0837565117	-5.6183377675	-1.9229050943
Si	-7.1426613563	-4.9191340658	0.3665820259
Si	-8.6173542997	-1.4447155724	-0.7674150889
Si	-5.4051205683	-5.9729266623	1.6331251871
Si	2.4102472185	-6.9495224758	2.2421855382
Si	4.5280918598	-6.3790360671	1.2768856801
Si	2.2894323600	-6.2439787884	4.5264284204
Si	2.4888057897	-3.1216535141	6.9386148999
Si	0.1732867613	-6.8165125790	5.4945581793
Si	-3.7330980803	-6.3266256384	5.1912082355
Si	-5.4679237338	-5.2725498454	3.9217026321
Si	-5.2652180031	-2.1493731225	6.3305126170
Si	-5.0012391225	0.2296868928	6.4188887320
Si	-6.7373070009	1.3301689714	5.1911969494
Si	-8.4157981914	1.6817365419	1.6477307091
Si	-6.4768436896	3.7098947691	5.2815797073
Si	-4.0448009669	6.6995610060	4.4506042126
Si	-0.1293470181	6.2114906899	4.7519629907
Si	-3.5814615742	7.6175530042	-0.0098412929
Si	-1.9028536945	7.2668114135	3.5360835267
Si	2.0121087266	6.7802302396	3.8383252252
Si	5.2299020953	2.2465369798	6.2123998287
Si	3.7869611815	5.7260732231	5.0536788390
Si	6.0034529280	5.6201275501	1.8510271538
Si	7.4220975144	2.1123351308	3.0420392331
Si	4.9233185850	-0.1265655673	6.1090449701
Si	7.1452927938	-0.2609159157	2.8781578694

Si	6.9520331530	-3.3944791475	0.4518678904
Si	7.2157073163	-1.0213475464	0.6057000116
Si	7.0431974203	-4.1336812157	-1.8253606511
Si	5.3723928686	-3.7789808371	-5.3767084500
Si	2.8041296172	-0.7496186691	7.0322636368
Si	0.3695336679	-3.7428894031	7.8670252631
Si	-3.5328454404	-3.2518086580	7.5622066588
Si	6.7779992635	-6.5107957089	-1.9702517077
Si	5.1215964610	-6.1600087304	-5.4736358315
Si	0.6880130967	4.2814023325	-8.8963108144
Si	0.8842063438	7.3651442835	-6.5197458552
Si	-6.6264877405	-4.7012121930	-6.3909218825
Si	-8.8290980605	-4.5706858074	-3.1867925780
Si	-5.8189904363	7.7625700004	3.2419322310
Si	-8.2213051383	4.8112785232	4.0632875458
Si	5.9287192363	6.3015901129	4.1458350271
Si	7.3546255584	2.8687614650	5.3110475522
Si	-3.7993956216	-5.6312729640	7.4816804179
Si	0.0547992863	-6.1162120441	7.7811327243
Si	-2.0625692153	-6.7111966711	8.7133810171
Si	0.9819504386	6.6492063380	-8.7951550893
Si	7.6706642882	5.2349749881	5.3831466791
Si	-8.7475983147	-5.2898753659	-5.4626989877
Si	-7.9482764920	7.1818149005	4.1551956229
Si	6.8726214453	-7.2245222393	-4.2464272862
H	-1.1209140701	-7.7390672512	-4.9127857127
H	0.5107500799	8.6248548550	0.3501814193
H	5.4258681663	3.2102507560	-4.7114018620
H	6.9149739929	-0.3001191004	-3.5715447105
H	-0.2081485599	-2.6336684720	-8.3492068073
H	-4.7582223148	4.4642181357	-5.4952459496

H	-6.9920927054	4.5954332334	-2.2449950988
H	-7.1921556239	1.4712691666	-4.6608526850
H	0.5565528600	-8.0842275231	-1.3698379119
H	-3.3530380005	-7.5978754784	-1.6752694143
H	-1.6796227969	-7.9511864173	1.8763907868
H	-8.2734783485	-2.1655702710	3.3797037813
H	-1.1597120114	0.1865452672	8.1606409572
H	-2.6277930023	3.6597570803	7.0230950134
H	1.2854630513	3.1781656631	7.3205705956
H	5.8186267059	-3.9222378890	4.4823918492
H	4.4622536724	-8.5757540033	-1.0729670204
H	8.3793763006	-3.7435807400	-2.4215282365
H	2.7889356654	-8.2236281081	-4.6077117606
H	6.7111287103	-3.3954441847	-5.9663588554
H	-2.5318650789	4.3361926930	-8.7400911618
H	3.7626944617	3.5500006589	-8.2469408613
H	3.9607258587	6.6645776317	-5.8444512131
H	-2.3371074447	7.4462419194	-6.3348974134
H	-5.0311109766	-7.2529151032	-5.2190616078
H	-7.3979797413	-1.6410215951	-7.0722474225
H	-7.2545687780	-7.1196091648	-1.9843414478
H	-9.6240987112	-1.5086506392	-3.8349940637
H	-6.7969784435	7.7115734123	0.1630896213
H	-9.2198203015	4.7339595364	0.9890922087
H	-4.1010538117	7.1267978940	5.9009245092
H	-6.5242340512	4.1511079283	6.7280284105
H	3.7128207439	6.1509919304	6.5040398677
H	5.1292248402	2.6862159043	7.6560516458
H	7.3296615151	5.9868292543	1.2477155283
H	8.7762324775	2.4658901503	2.4817056488
H	0.2996076320	-3.2874679996	9.3079071808

H	-0.0187394777	-8.3144093009	5.4209308182
H	-3.9079115287	-7.8270688488	5.1191569003
H	-3.5870854039	-2.7939574078	9.0032725956
H	-5.1458539016	-5.9906129985	8.0659839377
H	1.1609060391	-6.7846507642	8.5641537208
H	1.0641432759	8.8640919496	-6.4449954793
H	0.7475873028	3.8214762518	-10.3347639734
H	8.4462680951	2.1698773842	6.0893985896
H	6.1022503582	7.8013086871	4.2182560080
H	-10.1600919030	-4.9393545515	-2.5739980076
H	-6.5507563189	-5.1352033887	-7.8363837123
H	-5.6231747939	9.2603223974	3.2924281947
H	-9.5529422300	4.4242076888	4.6640941763
H	7.8705746324	-7.1738764372	-1.1652635305
H	5.1763513242	-6.5930759821	-6.9202604290
H	8.2076192055	-6.8628697521	-4.8453232511
H	6.7189654337	-8.7220212899	-4.3146801151
H	-8.0624446982	7.6618713909	5.5796633375
H	-9.0347527276	7.8469039715	3.3487605839
H	-2.1257488606	-6.3016286712	10.1627544551
H	-2.2464368612	-8.2060457969	8.6452621945
H	-0.0907392860	7.3355323235	-9.6016322532
H	2.3173697519	7.0202584557	-9.3880307760
H	7.6652917682	5.7011885824	6.8169032611
H	9.0100395672	5.5772384797	4.7816554679
H	-9.8475431771	-4.6339607545	-6.2574980993
H	-8.9454740219	-6.7823838820	-5.5338167155
H	-9.9490107265	-1.8225685728	-0.1577257556
H	-9.4244322092	1.6109185179	-1.4232042331
H	-9.7471883157	1.3024022295	2.2571664097
H	-8.4823567947	-5.2823183565	0.9671699238

H	-6.8094155600	-5.6339680744	4.5194955829
H	-6.6072789900	-2.5206546473	6.9215297499
H	-8.0769428519	0.9542108822	5.7847140319
H	-5.0532189636	0.6781762913	7.8630066675
H	3.3919381294	-6.9090775358	5.3191758177
H	3.5891454933	-3.8002208785	7.7235197581
H	6.0248991699	-0.8075404195	6.8902876291
H	5.6304351663	-7.0409477130	2.0720139960
H	8.0532045059	-4.0578811690	1.2484639051
H	8.2449712104	-0.9436928161	3.6620341855
H	8.5588110811	-0.6700633187	-0.0022937856
H	5.6356257169	6.3171113898	-2.3020259219
H	5.2378189239	0.0762524361	-7.1103451571
H	1.2693801357	-6.1014337512	-7.2227989406
H	3.6941581975	-3.1236338435	-8.0472738792
H	2.2184359215	0.3497843003	-9.1872412306
H	-2.6429765267	-5.6160969617	-7.5283948342
H	-4.1122245823	-2.1518360428	-8.6570696292
H	-1.6759164500	0.8388632838	-9.4908525731
H	-4.9641778718	1.3473352504	-7.9065289119
H	-4.5698948685	7.5790449144	-3.0848206572
H	-1.1602728809	8.9860409253	-3.1949756970
H	2.7254258941	8.5057834925	-2.8944729469
H	4.4046585738	8.1522664427	0.6609537776
H	-3.3890419650	9.1168046998	0.0547226685
H	-1.7173738756	8.7675888912	3.5842527874
H	2.1943600410	8.2813237091	3.8899033037
H	-0.1948882410	6.6416078136	6.2012100634
H	2.7297593190	-0.3003240272	8.4749159889
H	2.2152803669	-8.4469934508	2.1755219010
H	-5.5788889068	-7.4736935857	1.5674495054

S	7.5711480441	3.1998900416	-1.7663298719
C	9.1041820820	2.5331201920	-0.8846532237
H	8.8603543327	1.5804668256	-0.4123927342
H	9.8134714239	2.3259679251	-1.6931974036
C	9.7423958684	3.5135011099	0.0917720382
O	9.4259331741	4.7169427386	0.1671398231
N	10.7524643554	3.0076055786	0.8856520537
H	11.1820981152	3.7088179925	1.4767642743
C	11.1425778286	1.6142897224	1.0983724582
H	10.2675746988	0.9651348145	0.9910009348
H	11.4695523763	1.5144240435	2.1390438344
C	12.2847079606	1.1222757814	0.1659098974
H	11.9755364824	1.2625087443	-0.8779581963
H	13.1705878548	1.7445258306	0.3290732341
C	12.6050405528	-0.3309298435	0.4244053389
C	12.9656883559	-3.1672719116	0.9117847655
C	11.6730839767	-1.3433193437	-0.0995372910
C	13.6837096862	-0.7140174265	1.1591003873
C	13.9507222358	-2.1201885636	1.4628678320
C	11.8334181570	-2.6691438715	0.1224269543
H	10.8299759612	-0.9928435446	-0.6883922615
H	14.3916015109	0.0068073243	1.5556309676
H	11.1452316026	-3.4097051093	-0.2698205501
O	14.9301938705	-2.4783890300	2.1458696991
O	13.1389682828	-4.3798117555	1.1347881550

$Si_{165}H_{100}$-DOP^{red}

Si	-0.5481189243	-0.0057119132	-0.0293937338
Si	1.6304713408	0.5807126612	-0.8906737766
Si	-2.2575391938	1.0427517651	-1.3761775419
Si	-0.8274765029	-2.4010187689	-0.1025477542

Si	-0.7353602268	0.7641300191	2.2499756531
Si	-2.0690891938	0.2744149151	-3.6571796360
Si	-1.9830519891	3.4397708242	-1.3022958043
Si	-4.4369272753	0.4564208000	-0.5149813035
Si	-2.9147319148	0.1776588616	3.1119403437
Si	-0.4540777777	3.1607773735	2.3250279433
Si	1.8965538884	2.9777866297	-0.8149537331
Si	1.8224655036	-0.1858059185	-3.1715713935
Si	3.3456700526	-0.4626703787	0.4552548187
Si	-0.6373712708	-3.1692311643	-2.3837449181
Si	-3.0065528489	-2.9876164156	0.7588971842
Si	0.8824490193	-3.4467298723	1.2456791845
Si	0.9755891343	-0.2827383690	3.5956752583
Si	1.5426937278	-2.5830136570	-3.2443878775
Si	3.0628141664	-2.8626672109	0.3832647826
Si	0.6935296961	-2.6803945138	3.5272117636
Si	-3.1946479644	-2.2192372158	3.0400493556
Si	-4.7158322228	-1.9403684181	-0.5895361163
Si	-2.3482306091	-2.1223058527	-3.7311251373
Si	0.1102651739	0.8615333696	-4.5184123578
Si	0.1927656372	4.0281252789	-2.1671892902
Si	1.7271692865	3.7493654934	1.4650543339
Si	-2.1666577237	4.2090709936	0.9790837586
Si	3.1574693878	0.3059078407	2.7357829937
Si	-4.6246378832	1.2262651370	1.7656856097
Si	3.2514784342	-3.6348265049	-1.8968903348
Si	3.4529581495	2.7031347902	2.7988100718
Si	0.3836513228	3.2594930579	-4.4487542795
Si	-4.5291838299	-2.7104592706	-2.8715487238
Si	-1.4866481662	-3.2676153061	4.3903324306
Si	-4.3472627609	3.6240063454	1.8419483614

Si	2.9790009867	-6.0310166087	-1.9734863910
Si	5.4295247368	-3.0495807002	-2.7519419232
Si	1.7308244713	-3.3531196569	-5.5230421842
Si	-0.9135809396	-5.5631309101	-2.4596786894
Si	0.6065353822	-5.8406412640	1.1725110279
Si	4.7599170898	-3.9213219087	1.7341746402
Si	5.5300473213	0.1216876403	-0.4073649045
Si	3.9991309042	0.4035349846	-4.0287681695
Si	0.2972154136	0.0936918890	-6.7975719206
Si	-2.1615550229	-2.8906343133	-6.0104252800
Si	-3.2851249019	-5.3809910894	0.6860100720
Si	-4.8083360135	-5.1062677079	-2.9478949878
Si	-6.2407084657	-1.6659667169	-4.2175901576
Si	-6.8933761362	-2.5270697182	0.2691191845
Si	-5.3716422639	-2.8061546230	3.9001703756
Si	-6.8024814392	0.6413602508	2.6244874581
Si	-6.1477855659	1.5017912256	-1.8570278244
Si	-3.1019863178	0.9462233699	5.3889888408
Si	0.7888996106	0.4829867247	5.8737876586
Si	4.8498173951	-0.7522748129	4.0927683057
Si	5.6456634344	3.3098046670	1.9511004591
Si	3.2550920166	3.4688832580	5.0814132250
Si	-0.6421335755	3.9300629319	4.6014795343
Si	2.0079157719	6.1444309290	1.5365385405
Si	4.0612719575	3.5755123432	-1.6810226500
Si	2.5591431244	3.8479507464	-5.3075039442
Si	-1.3226283545	4.3069733939	-5.7981101598
Si	0.4734266291	6.4227541467	-2.0934845964
Si	-3.6909880966	4.4857737871	-2.6466430545
Si	-1.8907516901	6.6044913440	1.0548508891
Si	-4.5354880635	4.3953886363	4.1205763857

Si	-6.0600376364	4.6723435691	0.5005903326
Si	-3.7775668080	1.3196012808	-5.0028902899
Si	-1.6743830595	-2.5009265505	6.6704406417
Si	-1.7657765756	-5.6636931305	4.3209200440
Si	2.3945941517	-3.7331773354	4.8786663943
Si	5.6770411760	-0.6605957728	-2.6738509616
Si	4.2363337510	2.7919492303	-3.9458786378
Si	5.7537676518	2.5235093138	-0.3280567135
Si	0.2855167317	7.1458867099	0.1892944750
Si	-3.4735802390	3.7013261936	-4.9080800263
Si	-5.8426718079	3.8835041560	-1.7604298027
Si	-5.9304327228	0.7161305815	-4.1182176282
Si	-7.0436164173	-1.7468608538	2.5367427447
Si	-2.8185997050	3.3312269837	5.4251722351
Si	-1.3882068480	-0.1145477828	6.6973045072
Si	1.0760470086	2.8681768685	5.9027731615
Si	4.5451873933	-3.1323486559	3.9936505067
Si	-3.0898677817	-6.1088113667	-1.5954193030
Si	-1.5693395595	-6.3864648086	2.0369900048
Si	0.8010732971	-6.5704974110	-1.1079919131
Si	0.0158618358	-2.2911022381	-6.8300916742
Si	2.8074064671	6.2369223379	-5.2261772594
Si	-1.2404669785	7.4332722812	-3.4409753638
Si	-1.0184718304	6.6898113058	-5.7066882598
Si	-1.1067812338	3.5241323601	-8.0614740761
Si	2.7185890182	3.0710874008	-7.5755382883
Si	2.4711458497	0.6899775431	-7.6235162146
Si	-1.4131709447	1.1499281179	-8.1131640832
Si	4.3362083493	5.9595643466	-1.5940891966
Si	2.6466304114	6.9754802317	-2.9539702634
Si	4.1835808649	6.6960971422	0.6774724397

Si	4.1552913891	-0.3758077518	-6.2945542881
Si	-3.5677600742	0.5392674900	-7.2652897716
Si	-3.3922264558	6.8665338289	-2.5551859256
Si	-5.7585949717	7.0556079157	0.5966884337
Si	-8.2172241043	4.0732609943	1.3800553857
Si	-8.3042951280	0.9031435237	-0.9816478614
Si	-8.3973802741	-2.2692202309	-3.3390558063
Si	-6.0286903985	-2.4496931979	-6.4812228409
Si	-3.8729500165	-1.8344600544	-7.3248814787
Si	-2.4419689698	-5.2747663216	-6.0538550810
Si	1.4514372976	-5.7374015410	-5.5664586965
Si	3.9058928564	-2.7563800467	-6.3466234293
Si	3.1720837828	-6.7650093920	-4.2540503378
Si	4.6936964589	-7.0453480653	-0.6258132970
Si	-0.7234146355	-6.3002186512	-4.7373711044
Si	-4.6189421765	-5.8392221844	-5.2293600330
Si	-6.9852089788	-5.6578862976	-2.0865864019
Si	-7.1426374125	-4.9139114448	0.1844746500
Si	-8.5749868993	-1.4735960985	-1.0865422771
Si	-5.4587039979	-5.9359568609	1.5473878792
Si	2.3116410780	-6.8625799519	2.5219056062
Si	4.4649232321	-6.3016211293	1.6388326344
Si	2.0949226499	-6.1140127776	4.7857499221
Si	2.1846879067	-2.9510484770	7.1405790503
Si	-0.0566012509	-6.6790245480	5.6747767130
Si	-3.9415979222	-6.2153883985	5.1848450689
Si	-5.6217472965	-5.1929154858	3.8173900930
Si	-5.5290460826	-2.0279013670	6.1661101856
Si	-5.2752316639	0.3519126099	6.2176080754
Si	-6.9591566218	1.4191862051	4.8907631578
Si	-8.4849176351	1.6959491736	1.2709472100

Si	-6.7121957005	3.8004000666	4.9459164860
Si	-4.2569864985	6.7817044458	4.1640139897
Si	-0.3591331204	6.3139071599	4.6467167138
Si	-3.6056146374	7.6158131785	-0.2916625721
Si	-2.0804539632	7.3396833323	3.3333285132
Si	1.8161906817	6.8758834928	3.8161230623
Si	4.9490398006	2.4092785612	6.4175022004
Si	3.5427956554	5.8543404904	5.1268629290
Si	5.8945796269	5.6978172487	2.0262539282
Si	7.2817414686	2.2218737737	3.3433241338
Si	4.6446642349	0.0344007596	6.3539950693
Si	7.0034337387	-0.1523285648	3.2256976325
Si	6.9151152017	-3.3309122917	0.8627923229
Si	7.1751764863	-0.9576615633	0.9754370627
Si	7.1075014004	-4.1111143133	-1.3936321054
Si	5.5892116866	-3.8283768798	-5.0201011803
Si	2.4900764357	-0.5771522497	7.1973570393
Si	0.0310979521	-3.5637546605	7.9898530026
Si	-3.8499761116	-3.0978944247	7.4975376753
Si	6.8533784228	-6.4902801622	-1.5046023050
Si	5.3491632715	-6.2106481076	-5.0842743763
Si	1.0445141541	4.1380119089	-8.9170828201
Si	1.1315605512	7.2642092220	-6.5965915398
Si	-6.3411402346	-4.8234745575	-6.5505234727
Si	-8.6777628117	-4.6459063624	-3.4489964138
Si	-5.9800709551	7.8147138667	2.8577606611
Si	-8.4056824790	4.8708900559	3.6307212531
Si	5.7189485653	6.4253745358	4.3020454534
Si	7.1089111639	3.0231771299	5.5932390986
Si	-4.1088331817	-5.4789304760	7.4573182073
Si	-0.2748142524	-5.9390095746	7.9429325793

Si	-2.4280674290	-6.5215593658	8.7959597520
Si	1.3196703025	6.5091187542	-8.8543129475
Si	7.4063556666	5.3925865737	5.6413172353
Si	-8.5004770182	-5.4025692403	-5.7078735243
Si	-8.1419802794	7.2435372794	3.6956829089
Si	7.0390182837	-7.2562717591	-3.7582824577
H	-0.9000770191	-7.8022796130	-4.7675290461
H	0.4581878975	8.6495024479	0.2309288906
H	5.5903350945	3.1594473738	-4.5040352295
H	7.0392740483	-0.3190603198	-3.2341942111
H	0.1469562890	-2.7674090293	-8.2612385837
H	-4.5470783961	4.3542573543	-5.7519382595
H	-6.9114311382	4.5379072453	-2.6090784151
H	-6.9973905674	1.3757845092	-4.9651249718
H	0.6222607920	-8.0733232044	-1.1465956185
H	-3.2606901094	-7.6125594074	-1.6354248720
H	-1.7406224433	-7.8899825659	1.9907120772
H	-8.4065875971	-2.1059442217	3.0895710624
H	-1.5163151273	0.3657083538	8.1272556558
H	-2.9462233517	3.8028909124	6.8580233789
H	0.9515270988	3.3434898740	7.3348355746
H	5.6162472093	-3.7869319511	4.8395896536
H	4.5059210462	-8.5456552594	-0.6712555389
H	8.4642775337	-3.7323205899	-1.9474518371
H	2.9884390640	-8.2660866266	-4.2829963732
H	6.9514354371	-3.4561319153	-5.5630688299
H	-2.1823934994	4.1807413055	-8.8975637074
H	4.0866050357	3.4289961435	-8.1128115652
H	4.1752719048	6.5783265147	-5.7729121291
H	-2.0966230862	7.3345480490	-6.5488650242
H	-4.7882885883	-7.3420242982	-5.2587112266

H	-7.0946086411	-1.7802431017	-7.3194566338
H	-7.1442746918	-7.1617183851	-2.1273207061
H	-9.4544491128	-1.6025571600	-4.1907292448
H	-6.8279856463	7.7015438517	-0.2555306418
H	-9.2735175755	4.7308536275	0.5204518150
H	-4.3811190784	7.2397943330	5.6003609143
H	-6.8299666545	4.2714748886	6.3786619249
H	3.4046341657	6.3107958241	6.5625311463
H	4.7883609709	2.8857286054	7.8438282143
H	7.2445370189	6.0485821184	1.4690050503
H	8.6621915847	2.5422766870	2.8293324456
H	-0.1071059517	-3.0728318629	9.4138794482
H	-0.2389015277	-8.1797888371	5.6213939557
H	-4.1049022406	-7.7184056670	5.1320181930
H	-3.9733951682	-2.6100035397	8.9241338048
H	-5.4795496992	-5.8241620836	7.9930306654
H	0.8022022348	-6.5906060482	8.7793795726
H	1.3000052928	8.7655805970	-6.5425310787
H	1.1749995806	3.6431848377	-10.3395583257
H	8.1690022134	2.3483773956	6.4343304558
H	5.8792913886	7.9275268376	4.3573500750
H	-10.0338711132	-5.0006681089	-2.8836713260
H	-6.1977790652	-5.2919656499	-7.9805141955
H	-5.7913285295	9.3140779830	2.8900425365
H	-9.7605938524	4.4937825337	4.1853015066
H	7.9129810881	-7.1388808193	-0.6448148982
H	5.4696649312	-6.6724854835	-6.5186805781
H	8.4023960241	-6.9282621629	-4.3115560648
H	6.8700641945	-8.7539495189	-3.7995932598
H	-8.3081927858	7.7423177445	5.1084996117
H	-9.2016979123	7.8971928187	2.8455094117

H	-2.5617060399	-6.0684614833	10.2276569568
H	-2.6032970806	-8.0189385295	8.7666265772
H	0.2671618805	7.1718954199	-9.7061285391
H	2.6688356369	6.8805244287	-9.4154031930
H	7.3178255375	5.8843709389	7.0640856026
H	8.7739908350	5.7363138536	5.1093895348
H	-9.5683980083	-4.7578778947	-6.5542733210
H	-8.6977401889	-6.8959692602	-5.7689283640
H	-9.9299474949	-1.8401112984	-0.5219284902
H	-9.3618146680	1.5644060745	-1.8371667397
H	-9.8394204951	1.3253251649	1.8340703630
H	-8.5066306082	-5.2615330931	0.7387003978
H	-6.9882955948	-5.5388462031	4.3664142113
H	-6.8949512231	-2.3879269543	6.7083982115
H	-8.3219961449	1.0523239004	5.4362137152
H	-5.3971954620	0.8312495131	7.6473763263
H	3.1700405329	-6.7603336573	5.6309871303
H	3.2573859365	-3.6123781385	7.9772326535
H	5.7153995316	-0.6318740739	7.1894429339
H	5.5373448304	-6.9445089989	2.4898803287
H	7.9758671986	-3.9822970337	1.7208707665
H	8.0658853657	-0.8197605981	4.0716436333
H	8.5428460733	-0.6320927949	0.4150739837
H	5.6914354420	6.3017069451	-2.1599843318
H	5.5186437234	-0.0129258356	-6.8404867625
H	1.5809628709	-6.2002539690	-7.0010325448
H	4.0305108121	-3.2351064071	-7.7765709464
H	2.5957769688	0.2060373636	-9.0516970144
H	-2.3074825809	-5.7385833900	-7.4876508770
H	-3.7322782267	-2.3154767506	-8.7523814669
H	-1.2754056282	0.6626316218	-9.5388464083

H	-4.6380326513	1.2025668647	-8.1031143447
H	-4.4672159733	7.5129094778	-3.4002543668
H	-1.0617516377	8.9348753104	-3.3919466605
H	2.8047167510	8.4794710212	-2.9073677319
H	4.3321813686	8.2012741305	0.7201676037
H	-3.4219576910	9.1170016837	-0.2483259026
H	-1.9016540675	8.8416362791	3.3637303454
H	1.9884110611	8.3785571086	3.8491161154
H	-0.4910757131	6.7749189503	6.0815668886
H	2.3464464383	-0.0925939301	8.6231527892
H	2.1216659105	-8.3626411691	2.4747464743
H	-5.6214849949	-7.4393322800	1.5001708269
S	7.5882718302	3.2496386102	-1.4903308876
C	9.1035004803	2.5136368545	-0.6378781726
H	8.8427815919	1.5354131057	-0.2304885019
H	9.8156353875	2.3471312957	-1.4521714307
C	9.7419809216	3.4141577488	0.4145006420
O	9.3864671488	4.5931496675	0.6220396562
N	10.7796195036	2.8569617511	1.1275117886
H	11.2058819872	3.4998524225	1.7831164877
C	11.2321819784	1.4612245609	1.1425694924
H	10.3722579092	0.7919792750	1.0349639243
H	11.6488158636	1.2630386998	2.1361017544
C	12.2901982350	1.1038625275	0.0673941786
H	11.8973179643	1.3675821154	-0.9216842976
H	13.1834970901	1.7202482470	0.2266914717
C	12.6337767326	-0.3722208578	0.1102894885
C	13.1720175315	-3.1344933957	0.2877081922
C	11.8645912398	-1.3080811375	-0.6011841790
C	13.6870070952	-0.8425638738	0.9174858009
C	13.9491278529	-2.2066485294	0.9994757129

C	12.1287992156	-2.6796021419	-0.5148995980
H	11.0524323267	-0.9631556014	-1.2339527468
H	14.3008216041	-0.1379410367	1.4744828531
H	11.5411606668	-3.4054571892	-1.0642975191
O	14.9706377394	-2.7832510204	1.7647219747
H	15.5034783030	-2.1232739567	2.2431973405
O	13.4420025002	-4.4890657317	0.3771676608
H	14.2034797501	-4.6329189278	0.9767902698
